# Discovery of Primaquine–Indole Carboxamides with Cancer-Cell-Selective Antiproliferative Activity

**DOI:** 10.3390/molecules30193988

**Published:** 2025-10-04

**Authors:** Benjamin H. Peer, Jeremiah O. Olugbami, Dipak T. Walunj, Adegboyega K. Oyelere

**Affiliations:** 1 School of Chemistry and Biochemistry, Georgia Institute of Technology, 901 Atlantic Drive, Atlanta, GA 30332, USA; bhpeer@uw.edu (B.H.P.); jeremiah.olugbami@chemistry.gatech.edu (J.O.O.); dipak.walunj@chemistry.gatech.edu (D.T.W.); 2 Parker H. Petit Institute for Bioengineering and Bioscience, Georgia Institute of Technology, 315 Ferst Dr. NW, Atlanta, GA 30332, USA

**Keywords:** indole carboxylic acids, primaquine, cancer, androgen receptor, cytotoxicity, cell cycle progression, reactive oxygen species

## Abstract

Indole carboxylic acids are endogenous tryptophan metabolites that have demonstrated a variety of bioactivities, including anticancer effects. Specifically, indole acetic acid (IAA) elicits anticancer activity when combined with ultraviolet B or reactive oxygen species (ROS) generators. Primaquine (PQ) is an approved drug which elicits antimalarial activity through ROS generation. We investigated the effects of ICA, IAA, PQ, their combination and PQ–indole carboxamide conjugates on the viability of selected cancer cell lines. We identified PQ–indole carboxamide **2** which elicited more potent antiproliferative effects than PQ and ICA/PQ combination. Our data revealed that compound **2** derived a significant part of its antiproliferative effect from ROS generation.

## 1. Introduction

**Figure 1:**
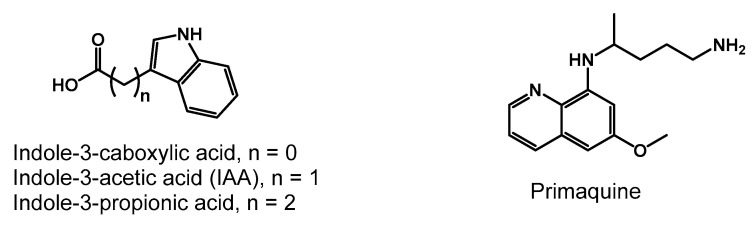
Structures of indole carboxylic acids derived from tryptophan metabolism and primaquine.

Metabolism of tryptophan in the human gastrointestinal tract by host and gut microbiota results in the generation of indole and several indole-based acids, including indole-3-carboxylic acid (ICA), indole-3-acetic acid (IAA), and indole-3-propionic acid (IPA) (Figure 1) [1,2,3]. IAA, a plant growth hormone, has been reported to enhance the efficacy of first-line chemotherapies in pancreatic cancer [4]. While IAA is highly tolerated in human body systems, it elicits an independent anticancer activity when combined with UVB or reactive oxygen species (ROS) generators such as horseradish peroxidase and phenothiazinium dyes [5,6,7,8,9]. Horseradish peroxidase-mediated activation of IAA involves oxidative mechanisms that result in toxic metabolites, including endoperoxide and singlet oxygen [6]. Combination treatments of IAA with other relatively benign FDA-approved oxidants and/or ROS inducers, which have demonstrated potential anticancer effects, could have enhanced clinical outcomes.

Primaquine (PQ) (Figure 1) is a commonly used antimalarial drug that kills malarial parasites, possibly due to the generation of ROS through the cycling of its hydroxylated metabolites [10,11,12,13]. PQ has also demonstrated weak anticancer effects, inhibiting the proliferation of cancer cells at high micromolar to millimolar IC_50_s [14,15,16]. Epidemiologically, there is an inverse correlation between malaria and multiple cancer types [17,18]. Hence, it is likely that the ROS-producing activity of PQ contributes to its anticancer effects. In addition, a variety of other mechanisms, including inhibition of endosomal trafficking and nuclear localization of epidermal growth factor receptor [14] and alleviation of P-glycoprotein-induced tumor resistance to chemotherapy [19,20], have been suggested to contribute to the anticancer activity of PQ.

In this study, we investigated the effects of the combination of IAA and PQ, in the context of standard combination therapy and carboxamide conjugate of primaquine–indole acetic acid (PQ-IAA **1**), on the viability of breast (MCF-7, MDA-MB-231, and MDA-MB-453), liver (Hep-G2), and prostate (DU-145 and LNCaP) cancer cell lines along with the Vero normal/control cell line. Although PQ is slightly more potent than PQ-IAA **1** and the IAA/PQ combination in some cell lines, we observed a disparity in the pattern of the antiproliferative effects of PQ, PQ-IAA **1**, and the IAA/PQ combination. Informed by this data, we synthesized a cohort of PQ–indole carboxamide conjugates incorporating indole acid analogs (compounds **2**–**7**). Evaluation of the antiproliferative activities of these compounds revealed that PQ–indole-3-carboxamide (PQ-ICA) **2** elicited enhanced potency with a preference for the prostate cancer cell line LNCaP. Subsequently, we synthesized and tested the antiproliferative activities of the enantiomers of **2** (compounds **8** and **10**) and **4** (compounds **9** and **11**). We surprisingly found that the racemic compounds **2** and **4** outperformed their individual enantiomers. Because the androgen receptor (AR) is vital to the viability of LNCaP cells, we conducted molecular docking analysis of the interaction of the R- and S-enantiomers of the disclosed PQ-indole compounds at the AR ligand binding domain (LBD) [21] and binding function 3 (BF3) sites [22] to obtain evidence for the involvement of AR interaction in their antiproliferative activities. Despite molecular docking predictions, which suggested that these compounds are better accommodated at the AR-LBD (Figure S3; Tables S1 and S2), evaluation of their representative examples in cell-free AR binding assay revealed that these compounds lacked AR-binding affinity (Figure S4). Additionally, we probed for the ROS-inducing effects of compound **2**, ICA, PQ, and the ICA/PQ combination in LNCaP cells. Our data revealed that **2** is a much more effective ROS inducer, generating higher levels of ROS in LNCaP cells when compared with hydrogen peroxide, ICA, PQ, and the ICA/PQ combination. Finally, we observed that LNCaP cells were dose-dependently arrested by **2** at both S and G2/M phases. Overall, these results suggest that **2** derived a significant part of its antiproliferative effect from ROS generation. However, the mechanism(s) of ROS generation activity of **2** is yet to be elucidated.

## 2. Results and Discussion

### 2.1. Synthesis

**Scheme 1:**
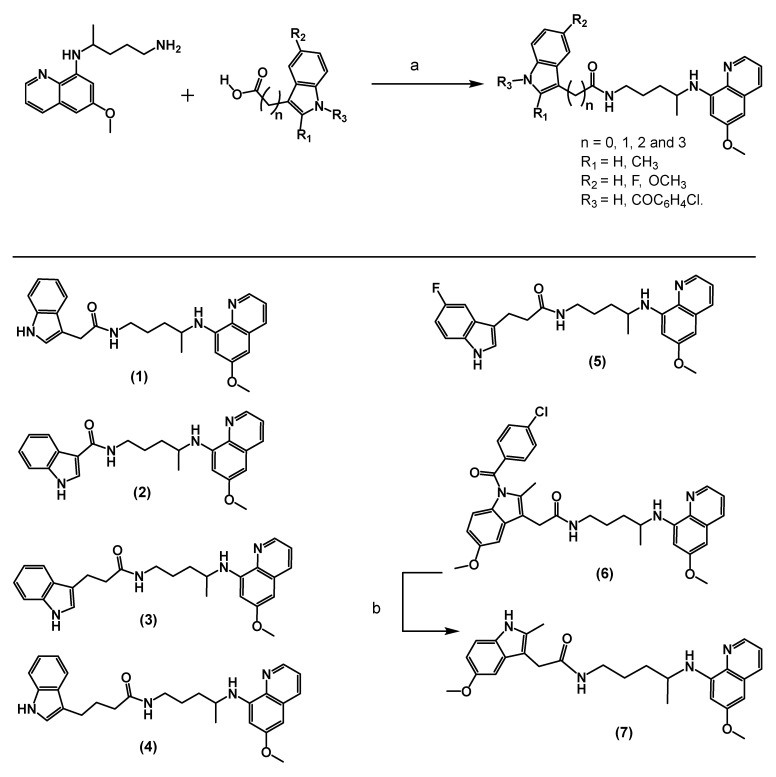
Synthesis of PQ–indole carboxamides **1–7**. Reagents and conditions: (a) EDCI.HCl, DMAP, CH_2_Cl_2_, rt, 15 h; (b) LiOH, THF: MeOH: H_2_O, rt, 15 h.

**Scheme 2:**
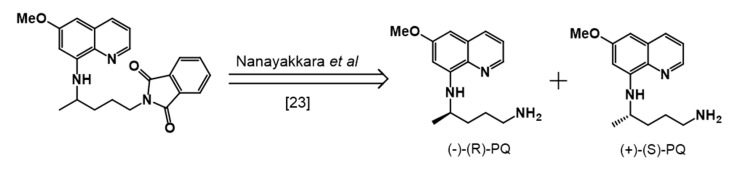

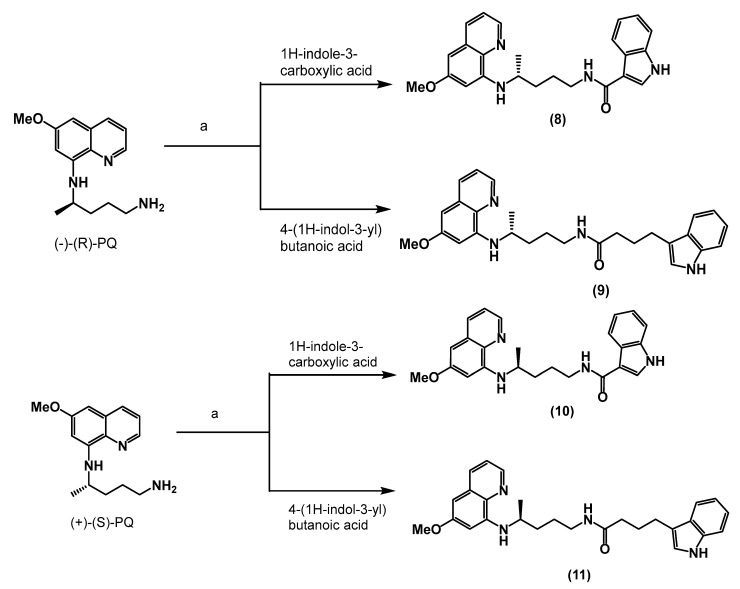
Synthesis of enantiomerically pure PQ–indole carboxamides **8–11**. Reagents and conditions: (a) EDCI.HCl, DMAP, CH_2_Cl_2_, rt, 15 h.

The synthesis of the designed PQ–indole carboxamide conjugates **1**–**6** involved a direct coupling of PQ with the corresponding indole carboxylic acid in dichloromethane (CH_2_Cl_2_) using EDCI and a catalytic amount of DMAP. A mild base treatment of compound **6** furnished PQ–indole carboxamide **7** (Scheme 1). The enantiomerically pure PQ–indole carboxamides **8**–**11** were similarly synthesized from (-)-(R)-primaquine and (+)-(S)-primaquine (Scheme 2). The requisite PQ enantiomers were obtained from the PQ racemate as described by Nanayakkara et al. [23]. The structures of the synthesized compounds were confirmed using ^1^H NMR, ^13^C NMR, and HRMS analyses (see Supplementary Materials).

### 2.2. Antiproliferative Activity of Primaquine–Indole Carboxamide

As single agents, IAA and ICA are not cytotoxic against the tested cell lines up to the maximum tested concentration (100 µM). In contrast, we observed that PQ displayed cell-line-dependent cytotoxic effects with IC_50_s ranging from high to low micromolar. Androgen receptor (AR)-expressing cell lines MDA-MB-453 (triple negative breast cancer cell line) and LNCaP (prostate cancer cell line) are more cytotoxic to PQ (Table 1, Figure S1). Others have shown evidence of cell-line-dependent cytotoxic effects of PQ [15,24]. Compared to PQ, PQ-IAA **1** is slightly more cytotoxic to most of the tested cell lines except for LNCaP and MDA-MB-253 cells. Interestingly, the IAA/PQ combination showed attenuated cytotoxicity against most of the tested cell lines, relative to PQ, except for MCF-7. Unlike PQ, the IAA/PQ combination is not toxic against LNCaP, although MDA-MB-453 is the most responsive cell line to this combination (Table 1). Informed by this disparity in the pattern of cell toxicity of PQ, PQ-IAA **1,** and the IAA/PQ combination, which seemed to favor PQ-IAA **1**, we synthesized and probed the antiproliferative effects of PQ–indole carboxamide conjugates **2**–**7** incorporating other indole acid analogs. We first investigated carboxamides derived from three unmodified indole carboxylic acids: ICA, IPA, and indole-4-butyric acid (IBA). Among these carboxamides, the ICA-derived compound **2** elicited the most potent, cancer-cell-selective antiproliferative effect, being more potent than PQ and **1** against all tested cancer cell lines. An exception is MDA-MB-453, against which **2** is about 1.7-fold less potent compared to PQ. Of the tested cell lines, LNCaP is the most sensitive to **2**, in a similar manner to the effect of PQ on this cell line. Also, we observed that **2** is more cytotoxic than the combination of ICA and PQ (Table 1). Subsequent modifications to the indole moiety on IAA and IPA did not translate to the enhancement of potency of the resulting compounds **3**–**7**. Moreover, we synthesized and tested the antiproliferative activities of the enantiomers of **2** (compounds **8** and **10**) and **4** (compounds **9** and **11**). Surprisingly, and due to reasons unclear to us, the enantiomerically pure PQ and compounds **8**–**11** were less active than their corresponding racemic compounds **2** and **4**. Racemic mixtures reconstituted from **8** and **10** and **9** and **11** elicited antiproliferative effects that nearly paralleled those of the corresponding compounds **2** and **4**, respectively (Table 2, Figure S2).

Intrigued by the LNCaP cell preference of PQ and **2**, we performed in silico docking using Autodock Vina (version 1.1.2) [25] to investigate the possibility of interaction of PQ, indole carboxylic acids, and representative indole carboxamides with AR, a nuclear receptor that is vital to the viability of LNCaP [26]. We conducted the molecular docking analysis at the AR ligand binding domain (LBD) [21] and binding function 3 (BF3) sites [22] using the R- and S-enantiomers of the disclosed PQ–indole compounds to obtain evidence for the involvement of AR interaction in their antiproliferative activities. Molecular docking predictions suggested that PQ–indole compounds are better accommodated at the AR-LBD (Figure S3, Tables S1 and S2). However, evaluation of selected PQ–indole compounds in a cell-free AR binding assay revealed that they lacked AR-binding activity (Figure S4).

### 2.3. Primaquine–Indole Carboxamide Generates Reactive Oxygen Species

**Figure 2:**
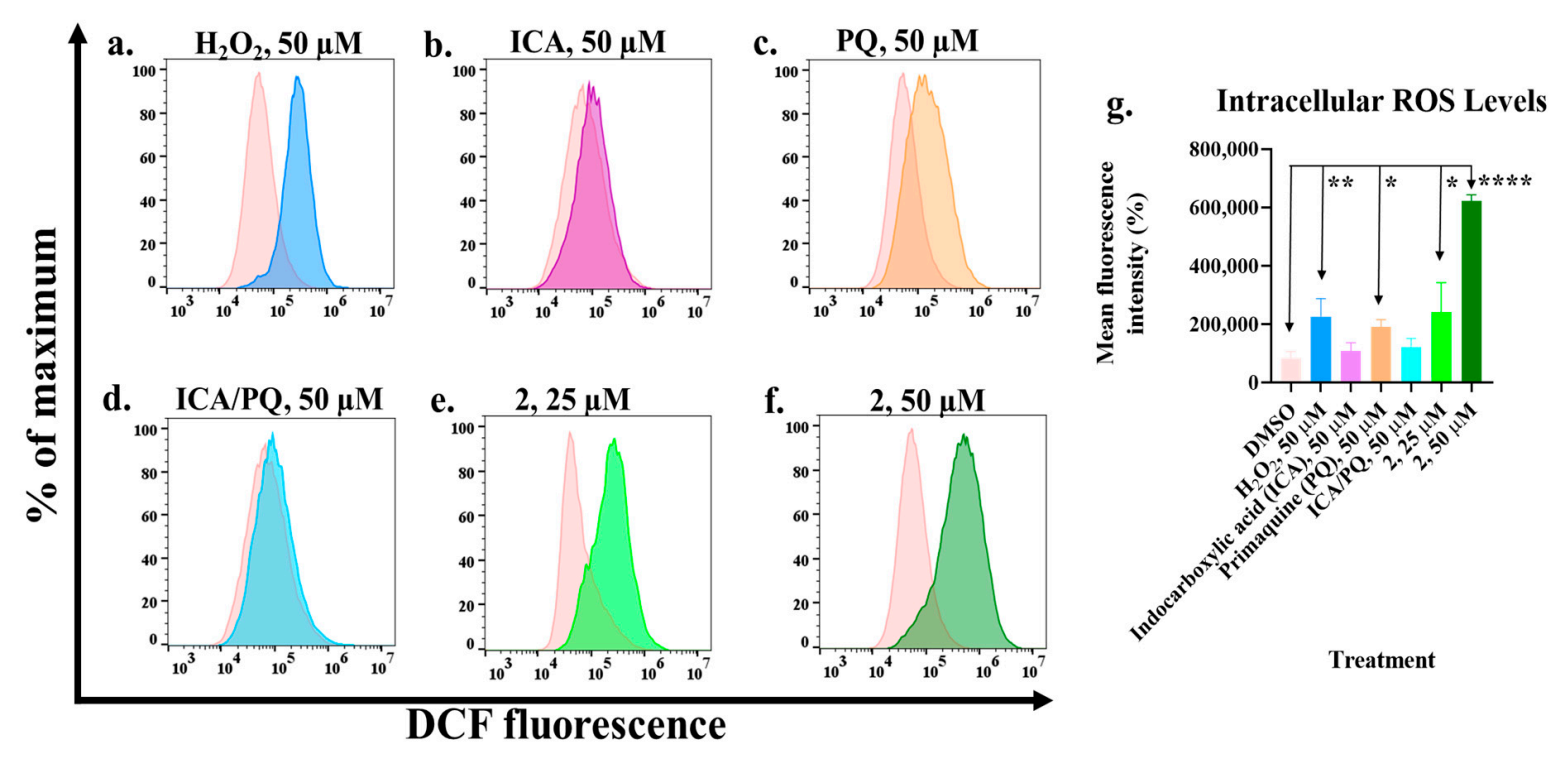
Changes in intracellular ROS levels, induced by the test agents, were measured in LNCaP cells, treated for 1 h, using DCFH-DA and flow cytometric analysis. Histogram representation of the fluorescence intensities of ROS levels in DMSO-treated cells in comparison with cells treated with 50 µM of hydrogen peroxide (H_2_O_2_, (**a**)), indole-3-carboxylic acid (ICA, (**b**)), primaquine (PQ, (**c**)), ICA/PQ (**d**), and **2** ((**e**,**f**), at 25 and 50 µM, respectively) along with their quantifications (**g**). * *p* < 0.05; ** *p* < 0.01; **** *p* < 0.0001.

The sustained proliferation, survival, and migration of cancer cells is dependent on the availability of relatively higher ROS levels in comparison to normal cells. However, ROS levels beyond a certain threshold can result in the induction of cancer cell death [27]. Since PQ is known to induce ROS in cells through the cycling of its hydroxylated metabolites [10,11,12,13,28], we probed for the ROS-inducing effects of **2**, ICA, PQ, and the ICA/PQ combination in LNCaP using hydrogen peroxide as a positive control. As shown in Figure 2, PQ, at a concentration of 50 µM, caused a significant induction of ROS, as previously reported [28]. Similarly, hydrogen peroxide also induced ROS in LNCaP cells at 50 µM [29]. Interestingly, we observed that **2** is a much more effective ROS inducer, generating higher levels of ROS in LNCaP cells when compared with hydrogen peroxide, ICA, PQ, and the ICA/PQ combination. The efficiency of ROS induction by **2** could partly explain its enhanced antiproliferative activities relative to PQ.

### 2.4. Cell Cycle Progression Is Modulated by Primaquine-Indole Carboxamide

**Figure 3:**
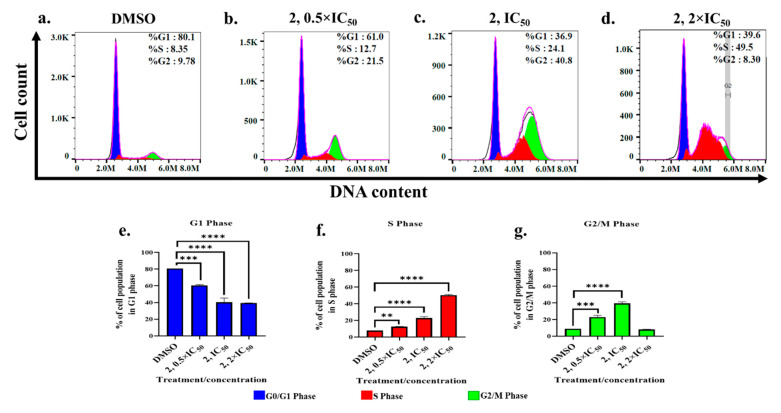
Modulation of cell cycle progression by compound **2** in LNCaP cells. Cell cycle distribution of both untreated (**a**) and treated (**b**–**d**) LNCaP cells was assessed by flow cytometric analysis. Unsynchronized LNCaP cells were treated with **2** for 24 h, subjected to PI/RNase staining, and the data obtained were analyzed by the Dean–Jett–Fox model using FlowJo software v. 10. From the distribution profiles (**e**–**g**), **2** caused a significant increase in accumulation of cells in both S and G2/M phases. The G2/M phase of **2** at 2×IC50 was manually adjusted. ** *p* < 0.01; *** *p* < 0.001; **** *p* < 0.0001.

Finally, we determined the effects of **2** on cell cycle progression. Progression through the cell cycle is normally regulated to ensure that cells with DNA damage are repaired before progressing to the next phase; however, if the damage is beyond repair, such cells are eliminated via programmed cell death mechanisms, such as apoptosis [30]. In the case of cancer cells, to allow for cellular proliferation, some cell cycle checkpoints are dysfunctional [31], and if there is an approach to halt progression through the cell cycle, then the possibility of inducing apoptosis increases. We observed that LNCaP cells were dose-dependently and significantly arrested by **2** at both S and G2/M phases (Figure 3), with the S-phase cell cycle arrest being more relevant at the highest concentration while the G2/M phase reverts to control levels.

## 3. Materials and Methods

### 3.1. Chemicals, Synthesis, and Charaterization

Anhydrous solvents and reagents were purchased from Sigma-Aldrich (St. Louis, MO, USA), Acros, VWR International (Radnor, PA, USA), or Thermo Fisher Scientific (Waltham, MA, USA) and were used without further purification. Analtech silica gel plates (60 F254, Miles Scientific, Newark, DE, USA) were utilized for analytical TLC, and Analtech preparative TLC plates (UV254, 2000 μm) [60 F254, Miles Scientific, Newark, DE, USA] were used for purification. Silica gel (200−400 mesh) was used in column chromatography. TLC plates were visualized using UV light, anisaldehyde, and iodine stains. NMR spectra were obtained on a Varian-Gemini 400 MHz and 700 MHz magnetic resonance spectrometer (Palo Alto, CA, USA). ^1^H NMR spectra were recorded in parts per million (ppm) relative to the residual peaks of CHCl_3_ (7.24 ppm) in CDCl_3_ or CHD_2_OD (4.78 ppm) in CD_3_OD or DMSO-*d5* (2.49 ppm) in DMSO-*d6*. ^13^C spectra were recorded relative to the solvent’s peaks with complete hetero decoupling. MestReNova (version 11.0) was used to process the original NMR “fid” files. High-resolution mass spectra were recorded at Georgia Institute of Technology’s Systems Mass Spectrometry Core facility.

### 3.2. Molecular Docking Study

Energy minimization of all structures was performed in ChemDraw 3D (Version 23.1). A previously reported AR homology model (PDB: 2AM9) was utilized to investigate binding to the AR-LBD [1]. In addition, another AR structure (PDB: 4HLW) was used to assess binding at the BF3 site. Molecular docking computations were conducted through AutoDock Vina via PyRx (Version 0.8), and the subsequent outputs were analyzed and imaged in PyMol (Version 2.6).

### 3.3. Cell Culture

Hep-G2 and DU-145 cancer cell lines were cultured in MEM (Corning, Glendale, AZ, USA) with 10% FBS. MCF-7 cells were cultured in 10% FBS DMEM without phenol red; MDA-MB-231 and Vero cells were cultured in DMEM (Corning, Glendale, AZ, USA) with phenol red, while LNCaP cells were cultured using RPMI-1640 supplemented with 10% FBS. All cell lines used were derived from American Type Culture Collection.

### 3.4. MTS Cell Viability Assay

Cells were plated into a 96-well plate at a density of 4500 cells/100 µL and allowed to adhere for 24 h prior to treatment. Subsequently, cells were exposed to varying concentrations of drugs for 72 h. All drug solutions were prepared in DMSO/DMEM, maintaining a DMSO concentration of 1%. The impact of these compounds on cell viability was assessed using the MTS assay, employing CellTiter 96 Aqueous One Solution from Promega (Madison, WI, USA), following the manufacturer’s instructions. IC_50_ values were determined using Prism GraphPad 8.

### 3.5. Quantification of Reactive Oxygen Species

This analysis was carried out based on the manufacturer’s instructions (Canvax Biotech, S.L., Valladolid, Spain) with some modifications. LNCaP cells (0.5 × 10^6^) were seeded in a 6-well plate for 24 h to allow cells to adhere. H_2_DCF-DA probe (15 µM final concentration) was added during treatment and incubated for 1 h. The cells were washed twice with 1 mL of ice-cold 1X phosphate-buffered saline (Corning, Glendale, AZ, USA), trypsinized, and the resulting cell suspension was transferred to microcentrifuge tubes. The cells were then centrifuged at 500× *g* for 5 min, after which the supernatant was carefully aspirated and discarded. For flow cytometry analysis, the tubes containing the stained cells were resuspended in 200 μL of growth medium and subsequently placed on ice. DCF fluorescence was detected by FACS, data were processed using FlowJo software, and the mean DCF fluorescence intensities were computed.

### 3.6. Cell Cycle Analysis

This analysis was carried out based on the manufacturer’s instructions (Canvax Biotech, S.L., Valladolid, Spain), also with some modifications. Briefly, cells (1 × 10^6^ cells/well in 2 mL of medium) were seeded in six-well plates and allowed to attach for 24 h before treatment for another 24 h. Subsequently, cells were dissociated from the substrate using a cell scraper (Avantor Inc., Radnor, PA, USA), and the cell suspension was centrifuged at 500× *g* for 5 min at 4 °C with the supernatant aspirated and discarded. Cells were then washed in 1 mL ice-cold PBS and centrifuged again with the supernatant aspirated and discarded. Next, nucleic acid labeling was initiated by initially fixing the cells with 1 mL of ice-cold 70% ethanol added dropwise to the cell pellet while vortexing, and the samples were stored on ice for at least 30 min. Thereafter, without disrupting the pellet, ethanol was carefully removed by centrifugation at 2500× *g* for 5 min at 4 °C, and the cells were subsequently washed in 1 mL PBS. Again, PBS was removed by centrifugation at 2500× *g* for 5 min, and then the cells were resuspended with 200 μL of the staining solution (freshly prepared by mixing PBS with propidium iodide and RNase A in a ratio of 50:1:1, respectively), which should be protected from exposure to light. In preparation for flow cytometry analysis (using CytoFLEX S Flow Cytometer (CytExpert v2.4), Beckman Coulter Life Sciences, Indianapolis, IN, USA), the tubes containing the stained cells were incubated in the dark for 30 min at 37 °C and finally placed on ice before analysis.

### 3.7. AR-Binding Assay

The androgen receptor competitive binding of compound 2 was assessed using the PolarScreen Androgen Receptor Competitor Assay Kit, Green (#A15880, Thermo Fisher Scientific Inc., Carlsbad, CA, USA), based on the manufacturer’s instructions.

## 4. Conclusions

Overall, while molecular docking analyses originally implicated the androgen receptor (AR), we showed in this study that PQ–indole carboxamides possess promising antiproliferative activities, but not by AR engagements based on negligible AR cell-free binding affinities. Our data revealed that compound **2**, the most potent among the PQ–indole carboxamides disclosed herein, derived a significant part of its antiproliferative effect from ROS generation. However, the mechanism(s) of ROS generation activity of **2** is yet to be elucidated.

## Figures and Tables

**Table 1:** Antiproliferative activities of primaquine–indole carboxamides.

Structure	IC_50_ (µM)						
	DU-145	LNCaP	Hep-G2	MCF-7	MDA-MB-231	MDA-MB-453	Vero
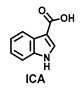	>100	>100	>100	>100	>100	>100	>100
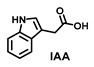	>100	>100	>100	>100	>100	>100	>100
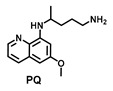	97.20 ± 5.6	29.50 ± 6.5	71.40 ± 12.4	90.92 ± 8.9	65.42 ± 2.3	13.72 ± 3.2	87. 55 ± 21.6
ICA + PQ	35.97	40.73 ± 1.1	82.57	NT	NT	NT	NT
IAA + PQ	>100	>100	82.65 ± 17.0	40.40 ± 8.5	44.94 ± 2.2	26.78 ± 5.7	>100
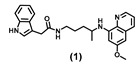	46.43 ± 9.3	48.67 ± 10.1	48.77 ± 1.3	48.27 ± 4.0	57.89	42.53 ± 2.5	91.50 ± 12.0
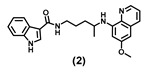	14.75 ± 3.8	11.90 ± 4.7	34.41 ± 4.3	43.68 ± 7.6	38.74 ± 3.4	23.83 ± 2.6	82.55 ± 15.7
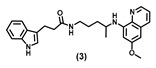	56.26 ± 11.7	33.47 ± 5.3	32.22 ± 1.6	67.10 ± 6.6	99.52 ± 1.6	43.54 ± 5.2	>100
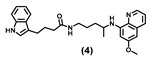	67.69 ± 11.0	25.93 ± 4.7	82.56 ± 13.1	90.55 ± 16.4	>100	30.41 ± 8.2	>100
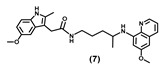	37.71 ± 4.0	32.90 ± 9.58	29.40 ± 10.0	42.51 ± 4.4	>100.00	33.57 ± 0.09	>100
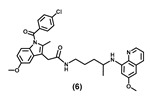	>100	>100	>100	>100	>100	>100	>100
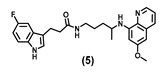	40.17 ± 4.0	24.28 ± 4.7	43.87 ± 3.8	48.77 ± 10.9	45.83 ± 5.1	36.20 ± 1.0	83.42 ± 2.3
Enzalutamide	21.26 ± 1.5	55.55 ± 7.9	>100	>100	>100	74.33 ± 22.7	>100

NT: not tested.

**Table 2:** Antiproliferative activities of enantiomerically pure PQ-carboxamides **8–11**.

Structure	IC_50_ (µM)						
	DU-145	LNCaP	Hep-G2	MCF-7	MDA-MB-231	MDA-MB-453	Vero
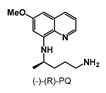	>100	>100	>100	>100	56.22 ± 7.1	23.45 ± 1.5	>100
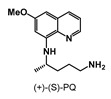	>100	>100	>100	>100	87.50 ± 12.5	28.19 ± 1.3	>100
(+)-(S)-PQ + (-)-(R)-PQ	41.37	52.39	57.95 ± 0.1	45.31 ± 5.5	38.46 ± 9.9	21.45 ± 2.6	>100
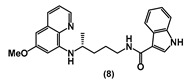	35.56 ± 1.8	40.38 ± 7.0	52.07	94.21 ± 5.0	53.09 ± 1.8	38.47 ± 4.6	>100
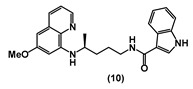	37.89 ± 2.5	32.09 ± 3.8	47.99 ± 2.7	78.51 ± 6.1	51.06 ± 4.7	16.06 ± 3.0	>100
**8** + **10**	25.15 ± 8.8	18.83 ± 1.7	29.96 ± 5.9	37.45 ± 8.8	26.31 ± 1.0	17.79 ± 0.9	>100
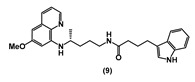	91.75 ± 11.7	24.75 ± 0.3	64.82 ± 5.6	99.72 ± 0.4	>100	31.82 ± 2.9	>100
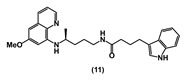	65.81 ± 5.4	21.29 ± 5.5	43.22 ± 2.6	98.50 ± 2.1	>100	19.30 ± 0.9	>100
**9** + **11**	49.80 ± 8.5	8.83 ± 2.0	26.90 ± 1.5	>50	>50	12.14 ± 0.8	>100

## Data Availability

The original contributions presented in this study are included in the article/Supplementary Materials. Further inquiries can be directed to the corresponding author.

## Supplementary Materials

The following supporting information can be downloaded at: https://www.mdpi.com/article/10.3390/molecules30193988/s1, Figure S1: Dose–response curves of the antiproliferative effects of the primaquine (PQ, a) and PQ–indole carboxamides (b–h) against the tested cell lines; Figure S2: Dose–response curves of the antiproliferative effects of the enantiomerically pure compounds **8**–**11** and racemic mixtures reconstituted from 8 and 10 and 9 and 11 against the tested cell lines; Figure S3: Images from molecular docking study of potential interaction of PQ–indole carboxamide with AR. (A) Docked indole moiety (in yellow; −6.2 kcal/mol) positioned next to the docked output of S-PQ (white; −4.7 kcal/mol). Note the overlap between the amine of primaquine and the amide of the indole moiety. (B) Docked orientation of **2R** (−8.6 kcal/mol) within the AR-LBD. The potential for hydrogen bonding between Met-74 and Thr-209 is detailed. (C) Docked output of **1S** (−6.9 kcal/mol) within the BF3 Site of AR (PDB: 4HLW). Hydrogen bonding was observed with Glu-837; Figure S4: Dose–response curves of the AR binding affinity of the PQ–indole carboxamides; Table S1: Docked scores of the PQ–indole carboxamides at AR-LBD compared to antiandrogen enzalutamide. Table S2: Docked scores of the PQ–indole carboxamides at the BF3 site compared to VPC-13789, an established AR-BF3 site binder.

## Abbreviations

PQ	Primaquine
ICA	indole-3-carboxylic acid
IAA	indole-3-acetic acid
IPA	indole-3-propionic acid
IBA	indole-4-butyric acid
AR	androgen receptor
LBD	ligand binding domain
BF3	binding function 3
