# Supplementary material for: Discovery of Primaquine–Indole Carboxamides with Cancer-Cell-Selective Antiproliferative Activity
Source: Molecules. 2025 Oct 4;30(19):3988. doi: 10.3390/molecules30193988 (PMC12526250; doi:10.3390/molecules30193988)

Discovery of Primaquine–Indole Carboxamides with Cancer-Cell-Selective Antiproliferative Activity

Benjamin H. Peer [1,†], Jeremiah O. Olugbami [1,†], Dipak T. Walunj [1,†] and Adegboyega K. Oyelere [1,2,*]

[1] School of Chemistry and Biochemistry, Georgia Institute of Technology, 901 Atlantic Drive, Atlanta, GA 30332, USA; bhpeer@uw.edu (B.H.P.); jeremiah.olugbami@chemistry.gatech.edu (J.O.O.); dipak.walunj@chemistry.gatech.edu (D.T.W.)

[2] Parker H. Petit Institute for Bioengineering and Bioscience, Georgia Institute of Technology, 315 Ferst Dr. NW, Atlanta, GA 30332, USA

* Correspondence: aoyelere@gatech.edu; Tel.: +1-404-894-4047

† These authors contributed equally to this work.

Supporting information

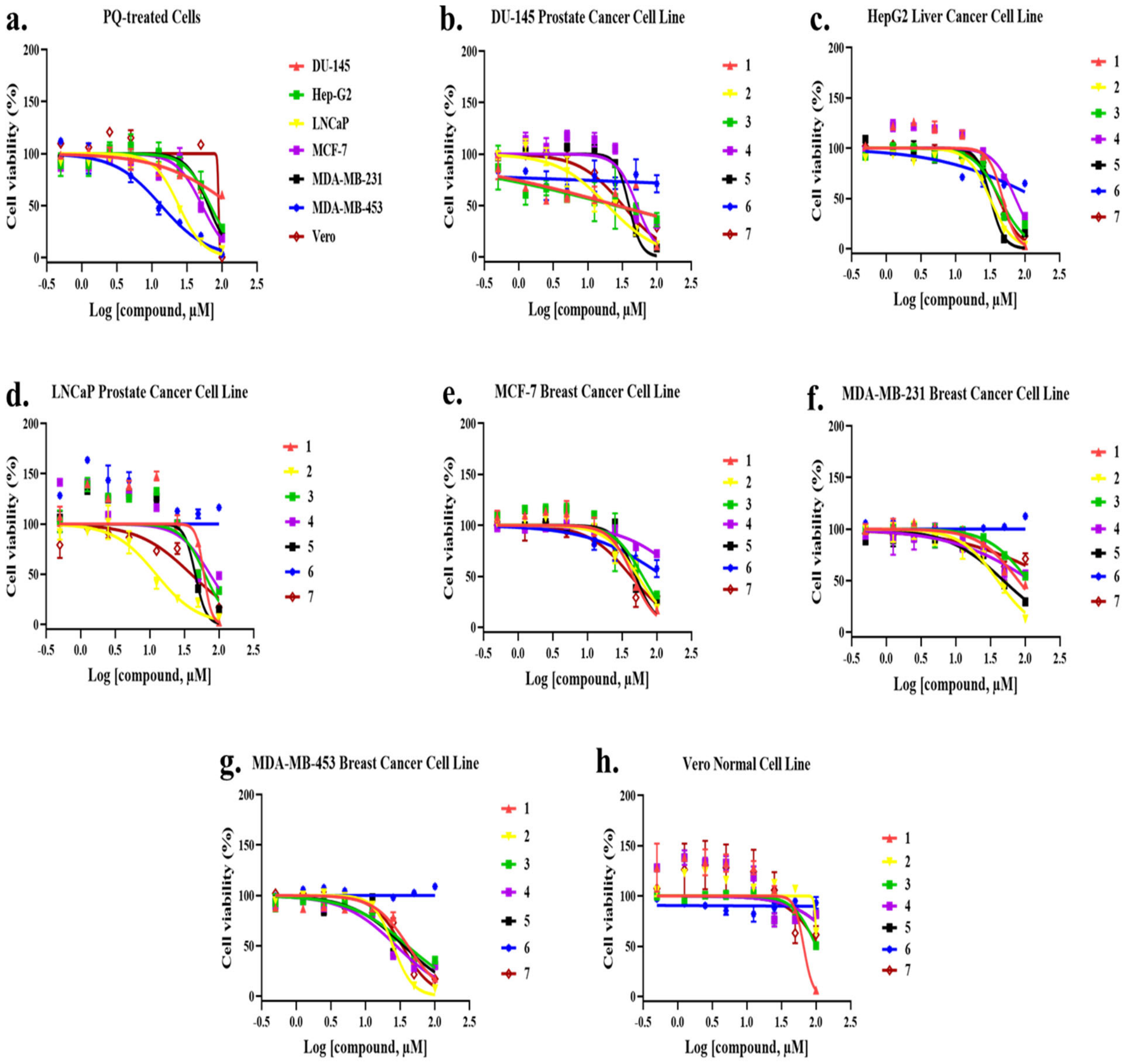

Figure S1: Dose response curves of the antiproliferative effects of the primaquine (PQ, a.) and PQ-indole carboxamides (b. – h.) against the tested cell lines.

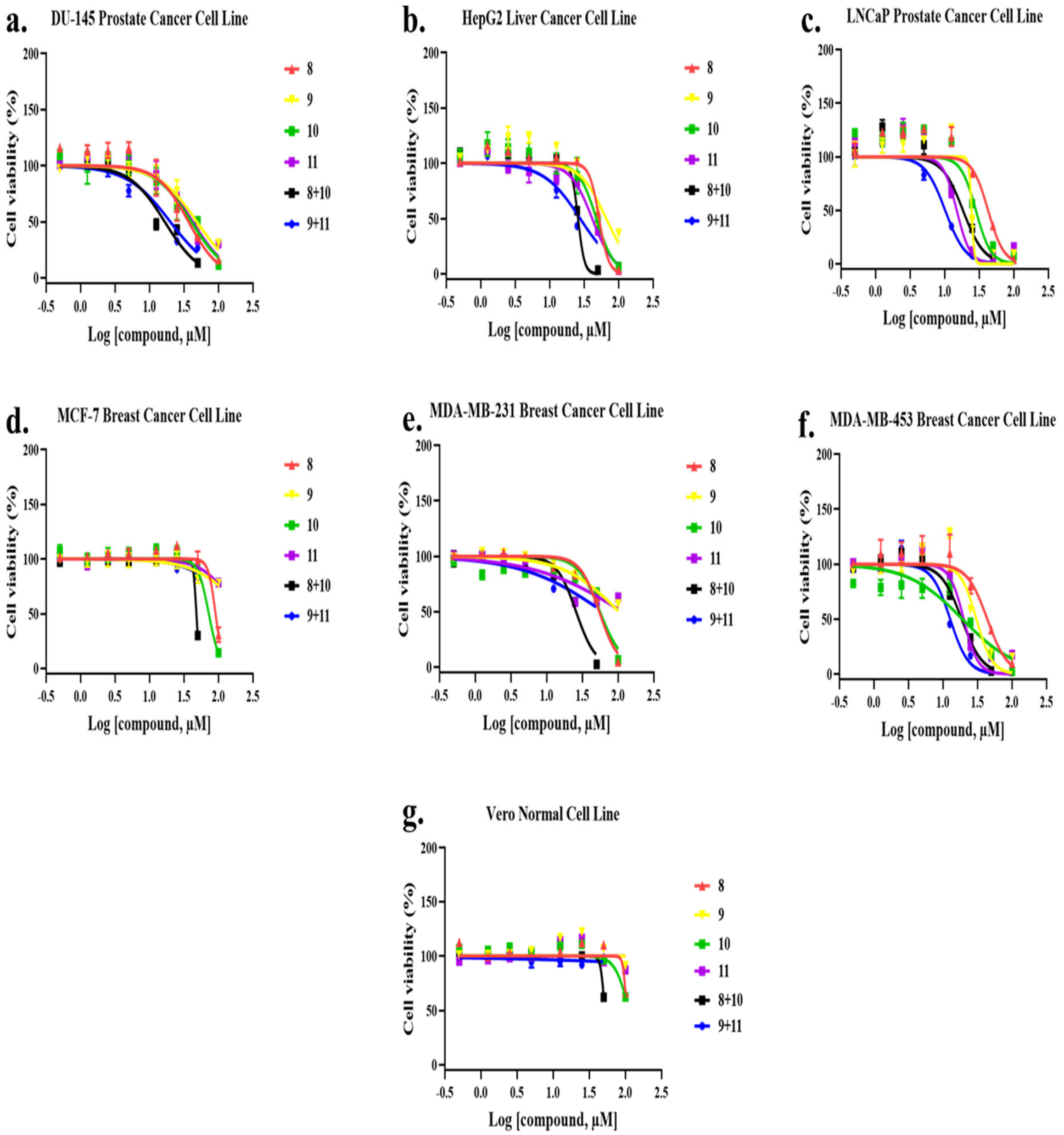

Figure S2: Dose response curves of the antiproliferative effects of the enantiomerically pure compounds **8-11** and racemic mixtures reconstituted from **8** and **10**, and **9** and **11** against the tested cell lines.

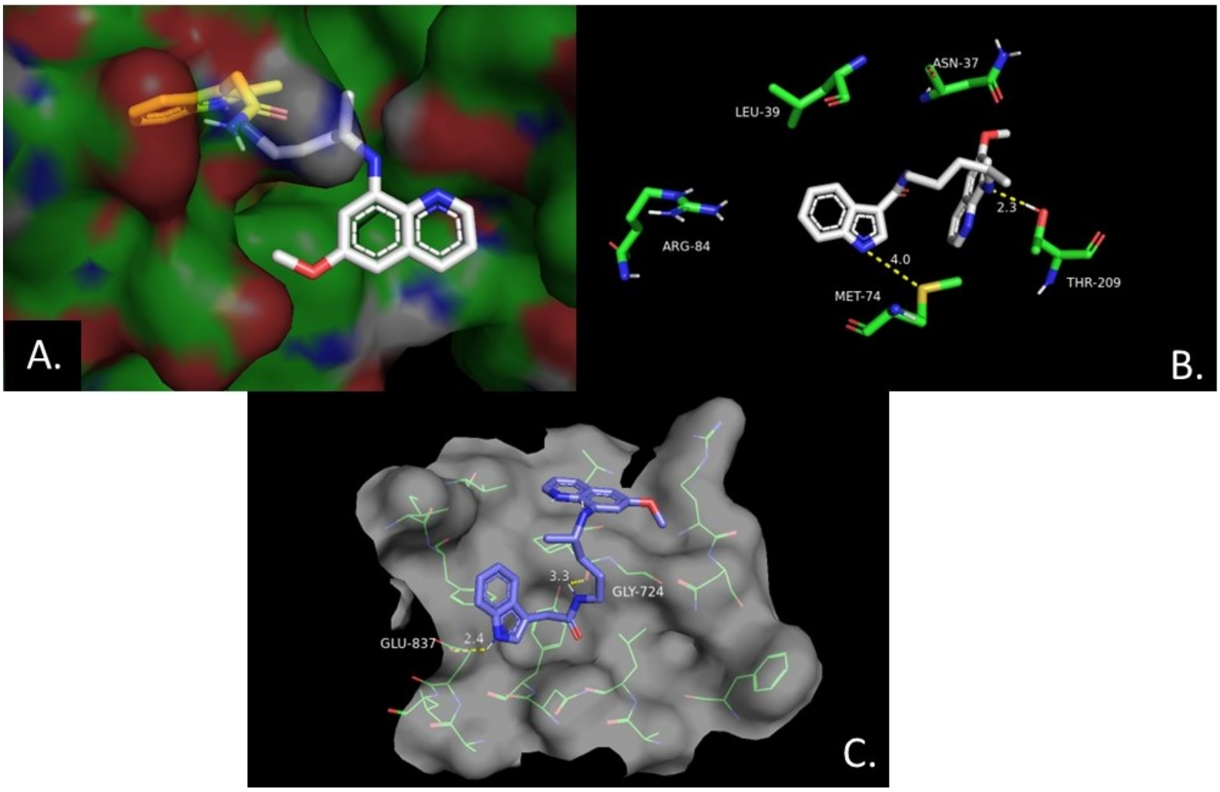

Figure S3: Images from molecular docking study of potential interaction of PQ-indole carboxamide with AR. A) Docked indole moiety (in yellow; -6.2 kcal/mol) positioned next to the docked output of S-PQ (white; -4.7 kcal/mol). Note the overlap between the amine of primaquine and the amide of the indole moiety. B) Docked orientation of **2R** (-8.6 kcal/mol) within the AR-LBD. Potential for hydrogen bonding between Met-74 and Thr-209 are detailed. C) Docked output of **1S** (-6.9 kcal/mol) within the BF3 Site of AR (PDB: 4HLW). Hydrogen bonding was observed with Glu-837.

Table S1: Docked scores of the PQ-indole carboxamides at AR-LBD compared to antiandrogen enzalutamide.

Compound	Binding affinity (kcal/mol)
Enzalutamide	-10.3
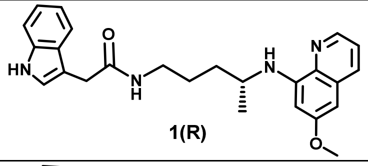 1(R)	-8.0
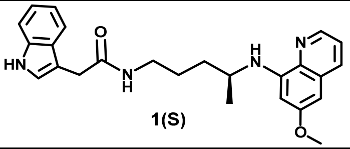 1(S)	-8.2
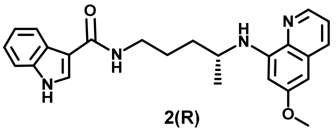 2(R)	-8.6
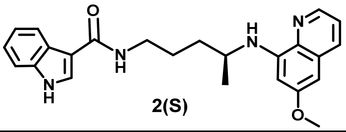 2(S)	-8.4
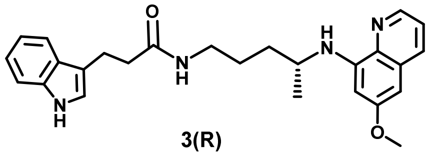 3(R)	-8.0
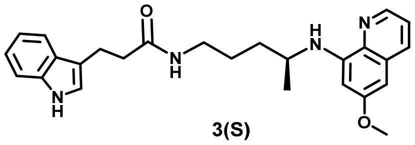 3(S)	-7.8
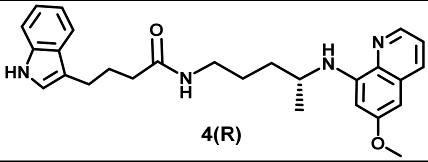 4(R)	-8.2
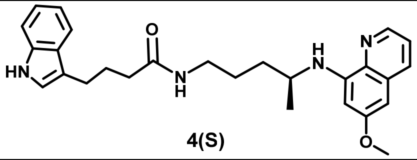 4(S)	-8.1
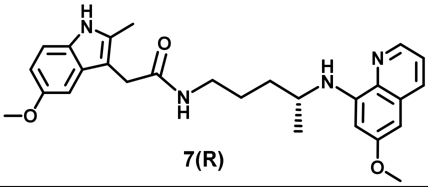 7(R)	-8.0

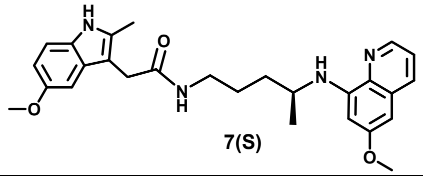 7(S)	-8.5

Table S2: Docked scores of the PQ-indole carboxamides at BF3 Site compared to VPC-13789, an established AR-BF3 site binder.

**Compound**	**Binding affinity (kcal/mol)**
VPC-13789	-7.3
**1S**	-6.9
**2S**	-6.7
**3S**	-6.8
**4S**	-6.3
**5S**	-6.8

Of the two building blocks, the indole fragment displayed greater binding affinity (-6.2 kcal/mol) compared to the PQ moiety (S-PQ, -4.7 kcal/mol) at the AR-LBD. However, the two building blocks are positioned in favorable orientation for connection between their primary amine and carboxylic acid moieties (Figure S3A). The PQ-indoles adopted docked poses having their indole and PQ moieties preserving the orientations of the corresponding building blocks with 2 displaying the most favorable affinity (-8.6 kcal/mol) (Figure S3A-B, Table S1). In contrast, the PQ-indole compounds are accommodated at the BF3 site less favorably, compared to AR-LBD, as evidenced by their dock scores (Figure S3C, Table S2). While each compound displayed comparable binding, 1 (-6.9 kcal/mol) nearly matched the computational score of VPC-13789, an established AR-BF3 site binder.

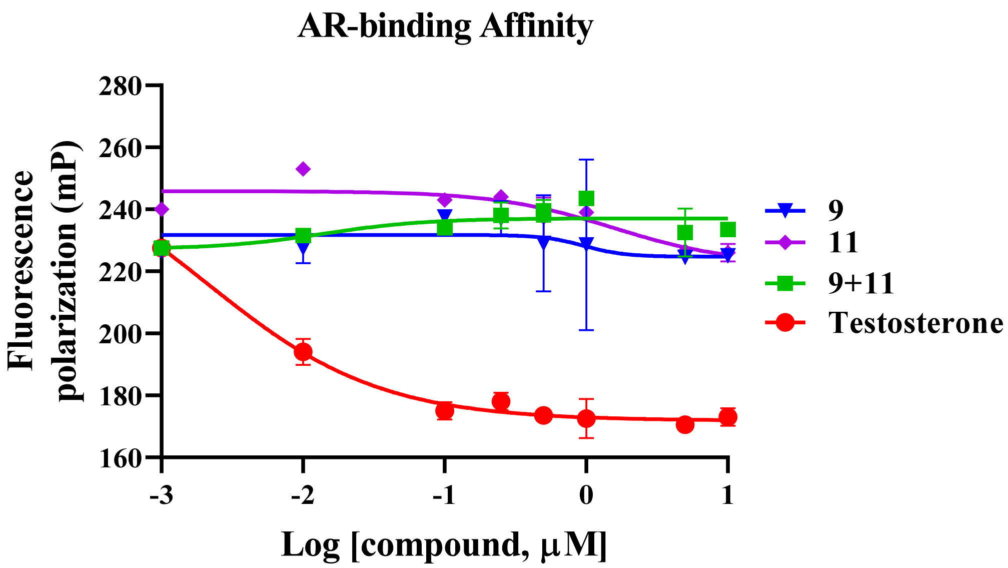

Figure S4: Dose response curves of the AR binding affinity of the PQ-indole carboxamides based on PolarScreen AR Competitor Assay, Green (Thermo Fisher Scientific Inc., Carlsbad, CA, USA).

Synthesis and Characterizations of Primaquine-Indole Carboxamides

General Procedure for the Synthesis of Primaquine-Indole Carboxamides

A mixture of Indole acid (1.1 mmol; 1.4 equiv.) and EDCI.HCl (1.7 mmol; 1.5 equiv.) in dry CH_2Cl_2 (10 mL) was stirred at 0 °C in an argon atmosphere for 30 min. Primaquine (1.1 mmol; 1 equiv.) and DMAP (0.11 mmol; 0.1 equiv.) were added, and the reaction was stirred at rt for 15 h. TLC indicated the completion of the reaction. The reaction was quenched with water (50 mL), extracted with CH_2Cl_2 (2×30 mL), and the combined organic layer was sequentially washed with

a saturated solution of $NaHCO_3$ and brine (30 mL). The organic layer was dried over Na_2SO_4 and then filtered. The solvent was removed using a rotary evaporator and the crude product was purified on silica gel column chromatography, eluting with CH_2Cl_2: MeOH (12:1), v/v, to afford solid the desired product.

2-(1H-indol-3-yl)-N-(4-((6-methoxyquinolin-8-yl)amino)pentyl)acetamide (1):

Following the general procedure 2-(1H-indol-3-yl) acetic acid (474 mg), EDCI.HCl (320 mg), Primaquine (500 mg) and DMAP (10 mg) gave **1 as** a solid (553 mg, 68.6 % yield). ^{1}H NMR (400 MHz, $CDCl_3$) δ 8.52 (dd, 1H), 8.29 (s, 1H), 7.92 (dd, *J* = 8.2, 1.7 Hz, 1H), 7.54 (d, *J* = 9.2 Hz, 1H), 7.37 (m, 1H), 7.31 (dd, *J* = 8.2, 4.2 Hz, 1H), 7.20 (m, 1H), 7.13 (d, *J* = 8.9 Hz, 1H), 7.08 (d, *J* = 2.4 Hz, 1H), 6.34 (d, J = 2.4 Hz, 1H), 6.22 (d, *J* = 2.4 Hz, 1H), 5.69 (s, 1H), 3.89 (s, 3H), 3.71 (s, 2H), 3.49 (m, 1H), 3.19 (m, *J* = 6.0 Hz, 2H), 1.49 (m, 4H), 1.19 (d, *J* = 6.3 Hz, 3H); ^{13}C NMR (176 MHz, $CDCl_3$) δ 159.4, 144.9, 144.4, 136.5, 135.3, 134.9, 130, 127, 123.9, 122.6, 121.9, 120, 118.7, 111.5, 108.9, 96.8, 91.7, 55.3, 47.8, 39.5, 33.7, 33.4, 26.2, 20.4. HRMS (EI) m/z Calcd. for $C_{25}H_{29}N_4O_2$ $[M+H]^+$: 417.2291, found 417.2246.

N-(4-((6-methoxyquinolin-8-yl)amino)pentyl)-1H-indole-3-carboxamide (2):

Following the general procedure 1H-indole-3-carboxylic acid (435 mg), EDCI.HCl (320 mg), Primaquine (500 mg) and DMAP (10 mg) gave **2** as a solid (589 mg, 75.8% yield). ^{1}H NMR (400 MHz, $CDCl_3$) δ 9.96 (s, 1H), 8.50 (dd, *J* = 4.3, 1.7 Hz, 1H), 7.94 (dd, *J* = 6.6, 2.9 Hz, 1H), 7.89 (dd, *J* = 8.3, 1.6 Hz, 1H), 7.62 (d, *J* = 2.9 Hz, 1H), 7.37 – 7.30 (m, 1H), 7.28 – 7.22 (m, 1H), 7.18 – 7.12 (m, 2H), 6.31 (d, *J* = 2.5 Hz, 1H), 6.27 (d, *J* = 2.5 Hz, 1H), 5.96 (s, 1H), 3.84 (s, 3H), 3.56 (m, 1H), 3.42 (m, 2H), 1.66 (m, 4H), 1.21 (d, *J* = 5.1 Hz, 3H); ^{13}C NMR (176 MHz, $CDCl_3$) δ 166

, 159.5, 145, 144.4, 136.5, 135.4, 135, 130, 128.1, 124.8, 122.8, 121.9, 121.5, 120, 112.2, 97, 91, 55.3, 47.9, 39.6, 34.1, 26.7, 20.6. HRMS (EI) m/z Calcd. for $C_{24}H_{27}N_4O_2$ $[M+H]^+$: 403.2134, found 403.2189.

3-(1H-indol-3-yl)-N-(4-((6-methoxyquinolin-8-yl)amino)pentyl)propanamide (3): Following the general procedure 3-(1H-indol-3-yl) propanoic acid (245 mg), EDCI.HCl (211 mg), Primaquine (240 mg) and DMAP (11 mg) gave **3** as a solid (338 mg, 84.9% yield). ^{1}H NMR (700 MHz, DMSO-*d6*) δ 10.74 (s, 1H), 8.54 (dd, *J* = 4.2, 1.6 Hz, 1H), 8.07 (d, *J* = 8.3 Hz, 1H), 7.83 (t, *J* = 5.6 Hz, 1H), 7.50 (d, *J* = 7.9 Hz, 1H), 7.42 (dd, *J* = 8.2, 4.2 Hz, 1H), 7.31 (d, *J* = 10.0 Hz, 1H), 7.04 (t, *J* = 6.9 Hz, 1H), 6.98 – 6.88 (m, 1H), 6.47 (d, *J* = 2.6 Hz, 1H), 6.26 (d, *J* = 2.6 Hz, 1H), 6.11 (d, *J* = 8.7 Hz, 1H), 3.81 (s, 3H), 3.64 – 3.55 (m, 1H), 3.10 – 3.00 (m, 2H), 2.92 – 2.85 (m, 2H), 2.41 (t, *J* = 8.0 Hz, 2H), 1.64 – 1.59 (m, 1H), 1.53 – 1.43 (m, 3H), 1.19 (d, *J* = 6.3 Hz, 3H). ^{13}C NMR (176 MHz, DMSO-*d6*) δ 171.6, 159, 149.6, 144.6, 144.2, 136.2, 134.8, 134.5, 129.5, 127., 123.9, 122., 120.8, 118., 113.9, 111.2, 96.1, 91.6, 54.9, 47, 38.4, 36.3, 33.4, 26, 20.9, 20.2. HRMS (EI) m/z Calcd. for $C_{26}H_{31}N_4O_2$ $[M+H]^+$: 431.2447, found 431.2471.

4-(1H-indol-3-yl)-N-(4-((6-methoxyquinolin-8-yl)amino)pentyl)butanamide (4)

Following the general procedure 4-(1H-indol-3-yl) butanoic acid (320 mg), EDCI.HCl (320 mg.), Primaquine (500 mg) and DMAP (10 mg) gave **4** as a solid (561 mg, 65.3 % yield). ^{1}H NMR (400 MHz, $CDCl_3$) δ 8.52 (dd, *J* = 4.3, 1.7 Hz, 1H), 8.13 (s, 1H), 7.93 (dd, *J* = 8.2, 1.7 Hz, 1H), 7.57 (d, *J* = 7.9 Hz, 1H), 7.34 – 7.28 (m, 2H), 7.19 – 7.13 (m, 1H), 7.08 (t, *J* = 8.0 Hz, 1H), 6.95 (d, *J* = 2.3 Hz, 1H), 6.34 (d, *J* = 2.6 Hz, 1H), 6.28 (d, *J* = 2.5 Hz, 1H), 5.40 (s, 1H), 3.88 (s, 3H), 3.61 (s, 1H), 3.25 – 3.12 (m, 2H), 2.77 (t, *J* = 7.7 Hz, 2H), 2.16 (t, *J* = 7.3 Hz, 2H), 2.06 – 1.98 (m, 2H),

1.60 (t, J = 15.3 Hz, 2H), 1.28 (d, J = 6.3 Hz, 3H).1.25 (m, 2H). ^{13}C NMR (101 MHz, $CDCl_3$) δ 173.1, 159.5, 145 144.4, 136.4, 135.4, 130, 127.5, 122, 121.9, 121.7, 119.2, 118.9, 115.6, 111.2, 55.3, 47.9, 39.5, 36.2, 34, 26.3, 26, 24.6, 20.6. HRMS (EI) m/z Calcd. for $C_{27}H_{33}N_4O_2$ [M+H]$^+$: 445.2604, found 445.2637.

3-(5-fluoro-1H-indol-3-yl)-N-(4-((6-methoxyquinolin-8-yl)amino)pentyl)propanamide (5): Following the general procedure 3-(5-fluoro-1H-indol-3-yl) propanoic acid (320 mg), EDCI.HCl (320 mg), Primaquine (500 mg) and DMAP (10 mg) gave **5** as a solid (495 mg, 55.8% yield). ^{1}H NMR (700 MHz, $CDCl_3$) δ 9.25 (s, 1H), 8.60 (dd, J = 4.2, 1.7 Hz, 1H), 7.99 (dd, J = 8.4, 1.9 Hz, 1H), 7.36 (dd, J = 8.2, 4.2 Hz, 1H), 7.23 – 7.10 (m, 2H), 7.05 (d, J = 2.3 Hz, 1H), 6.91 – 6.79 (m, 1H), 6.37 (d, J = 2.5 Hz, 1H), 6.26 (d, J = 2.5 Hz, 1H), 5.80 (s, 1H), 5.26 (s, 1H), 3.89 (s, 3H), 3.51 – 3.42 (m, 1H), 3.34 – 3.23 (m, 1H), 3.09 – 2.99 (m, 2H), 2.48 (m, 2H), 1.42 – 1.18 (m, 6H), 1.12 (dd, J = 6.3, 2.0 Hz, 3H); ^{13}C NMR (176 MHz, $CDCl_3$) δ 172.5, 159.6, 157.1, 144.9, 135.4, 133.1, 127.4, 124.5, 122.1, 114.5, 112.1, 110.1, 103.4, 97.1, 91.9, 55.3, 47.9, 39.2, 37.6, 34.4, 26.5, 21.6, 20.4. HRMS (EI) m/z Calcd. for $C_{26}H_{30}FN_4O_2$ [M+H]$^+$: 449.2353, found 449.2352.

2-(1-(4-chlorobenzoyl)-5-methoxy-2-methyl-1H-indol-3-yl)-N-(4-((6-methoxyquinolin-8-yl)amino)pentyl)acetamide (6) :

Following the general procedure 2-(5-methoxy-2-methyl-1H-indol-3-yl) acetic acid (360 mg), EDCI.HCl (211 mg), Primaquine (240 mg) and DMAP (11mg) gave **6** as a solid (420 mg, 75.8% yield). ^{1}H NMR (700 MHz, $CDCl_3$) δ 8.48 (dd, J = 4.2, 1.6 Hz, 1H), 7.89 (dd, J = 8.2, 1.7 Hz, 1H),

7.59 (d, *J* = 6.6 Hz, 2H), 7.43 (d, *J* = 8.5 Hz, 2H), 7.28 (dd, *J* = 8.2, 4.1 Hz, 1H), 6.87 (d, *J* = 2.6 Hz, 1H), 6.83 (d, *J* = 9.0 Hz, 1H), 6.67 (d, *J* = 2.5 Hz, 1H), 6.31 (d, *J* = 2.6 Hz, 1H), 6.22 (d, *J* = 2.6 Hz, 1H), 5.92 (d, *J* = 8.5 Hz, 1H), 5.71 (t, *J* = 6.0 Hz, 1H), 3.86 (s, 3H), 3.76 (s, 3H), 3.61 (s, 2H), 3.57 – 3.51 (m, 1H), 3.28 – 3.18 (m, 2H), 2.34 (s, 3H), 1.62 – 1.56 (m, 2H), 1.55 – 1.49 (m, 2H), 1.22 (d, *J* = 6.3 Hz, 3H). ^{13}C NMR (176 MHz, $CDCl_3$) δ 169.9, 168.3, 159.4, 156.3, 144.9, 144.4, 139.5, 136.3, 135.3, 134.8, 133.6, 131.2, 130.9, 130.4, 129.2, 121.9, 115.2, 112.4, 100.8, 96.8, 91.7, 55.7, 55.2, 47.8, 39.6, 33.9, 32.3, 26.4, 20.6, 13.3. HRMS (EI) m/z Calcd. for $C_{34}H_{36}ClN_4O_4$ $[M+H]^+$: 599.2425, found 599.2463.

2-(5-methoxy-2-methyl-1H-indol-3-yl)-N-(4-((6-methoxyquinolin-8-yl)amino)pentyl)acetamide (7):

The compound **6** (100 mg) was dissolved in 16 mL of THF: MeOH (1:1) 0 °C and subsequently 1M solution of LiOH (8 mL) was added, and reaction mixture was stirred at rt for 15 h. The completion of the reaction was indicated by TLC. The reaction was quenched with water (20 mL), extracted with CH_2Cl_2 (2×20 mL), and the combined organic layer was sequentially washed with a brine solution (30 mL). The organic layer was dried over Na_2SO_4 and then filtered. The solvent was removed using a rotary evaporator that gave **7** as a solid (74 mg, 97.3% yield). ^{1}H NMR (700 MHz, $CDCl_3$) δ 8.50 (dd, *J* = 4.2, 1.6 Hz, 1H), 8.21 (s, 1H), 7.91 (dd, *J* = 8.2, 1.7 Hz, 1H), 7.29 (dd, *J* = 8.3, 4.2 Hz, 1H), 7.15 (d, *J* = 8.6 Hz, 1H), 6.85 (d, *J* = 2.5 Hz, 1H), 6.77 (dd, *J* = 8.7, 2.4 Hz, 1H), 6.33 (d, *J* = 2.5 Hz, 1H), 6.21 (d, *J* = 2.5 Hz, 1H), 5.90 (s, 1H), 5.72 (t, *J* = 6.0 Hz, 1H), 3.88 (s, 3H), 3.76 (s, 3H), 3.61 (m, 2H), 3.50 (m, *J* = 6.2 Hz, 1H), 3.19 (q, *J* = 6.9 Hz, 2H), 2.30 (s, 3H), 1.56 – 1.49 (m, 2H), 1.48 – 1.42 (m, 2H), 1.20 (s, 3H). ^{13}C NMR (176 MHz, $CDCl_3$) δ 171.6, 159.4, 154.4, 144.9, 144.4, 135.4, 134.9, 134.1, 130.4, 129.9, 128.7, 121.9, 111.6, 111.4,

104.7, 99.8, 96.8, 91.7, 55.9, 55.3, 47.8, 39.4, 33.8, 32.3, 26.3, 20.4, 11.7. HRMS (EI) m/z Calcd. for $C_{27}H_{33}N_4O_3$ $[M+H]^+$: 461.2553, found 461.2589.

(R)-N-(4-((6-methoxyquinolin-8-yl)amino)pentyl)-1H-indole-3-carboxamide (8): Following the general procedure 1H-indole-3-carboxylic acid (43.5 mg), EDCI.HCl (32 m g), dry CH_2Cl_2 (2 mL), R-Primaquine (50 mg) and DMAP (1 mg) gave **8** as a solid (53 mg, 67.9 % yield). ^{1}H NMR (700 MHz, $CDCl_3$) δ 8.52 (dd, *J* = 4.2, 1.6 Hz, 1H), 7.92 (d, *J* = 1.7 Hz, 2H), 7.72 (d, *J* = 2.8 Hz, 1H), 7.41 (d, *J* = 6.6 Hz, 1H), 7.30 (dd, *J* = 8.2, 4.2 Hz, 1H), 7.25 – 7.20 (m, 2H), 6.33 (s, 1H), 6.02 (s, 1H), 3.87 (s, 3H), 3.69 (s, 1H), 3.58 –3.49 (m, 2H), 1.84 – 1.75 (m, 4H), 1.32 (d, *J* = 6.5 Hz, 3H). ^{13}C NMR (176 MHz, $CDCl_3$) δ 165.3, 159.5, 145.1, 144.4, 136.3, 134.9, 130, 127.7, 124.7, 123, 121.7, 120.1, 111.9, 96.9, 91.9, 55.3, 48, 39.6, 34.2, 29.8, 26.8, 20.7. HRMS (EI) m/z Calcd. for $C_{24}H_{27}N_4O_2$ $[M+H]^+$: 403.2134, found 403.2171.

(R)-4-(1H-indol-3-yl)-N-(4-((6-methoxyquinolin-8-yl)amino)pentyl)butanamide (9): Following the general procedure 4-(1H-indol-3-yl) butanoic acid (32 mg), EDCI.HCl (32 mg), dry CH_2Cl_2 (2 mL), R-Primaquine (50 mg) and DMAP (1 mg) gave **9** as a solid (63 mg, 73.3% yield). ^{1}H NMR (700 MHz, $CDCl_3$) δ 8.52 (dd, *J* = 4.2, 1.6 Hz, 1H), 8.16 (s, 1H), 7.93 (dd, *J* = 8.2, 1.6 Hz, 1H), 7.57 (dd, *J* = 7.8, 1.1 Hz, 1H), 7.32 (d, *J* = 8.2 Hz, 1H), 7.30 (dd, *J* = 8.2, 4.2 Hz, 1H), 7.18 – 7.14 (m, 1H), 7.08 (t, *J* = 8.0 Hz, 1H), 6.94 (d, *J* = 2.4 Hz, 1H), 6.34 (d, *J* = 2.5 Hz, 1H), 6.29 (d, *J* = 2.5 Hz, 1H), 5.98 (s, 1H), 5.41 (t, *J* = 5.8 Hz, 1H), 3.88 (s, 3H), 3.61 (s, 1H), 3.48 (s, 1H), 3.25 – 3.16 (m, 2H), 2.77 (t, *J* = 7.3 Hz, 2H), 2.18 – 2.13 (m, 2H), 2.05 – 1.97 (m, 2H), 1.68 – 1.54 (m, 4H), 1.28 (d, *J* = 6.3 Hz, 3H). ^{13}C NMR (176 MHz, $CDCl_3$) δ 173.1, 159.5, 145, 144.4,

136.4, 135.4, 135, 130, 127.6, 121.9, 121.7, 119.2, 118.9, 115.6, 111.2, 96.9, 91.8, 55.3 47.9, 39.5, 36.3, 34.1, 26.3, 26, 24.6, 20.7 HRMS (EI) m/z Calcd. for $C_{27}H_{33}N_4O_2$ $[M+H]^+$: 445.2604, found 445.2621.

(S)-N-(4-((6-methoxyquinolin-8-yl)amino)pentyl)-1H-indole-3-carboxamide (10): Following the general procedure 1H-indole-3-carboxylic acid (43.5 mg), EDCI.HCl (32 mg), dry CH_2Cl_2 (2 mL), S-Primaquine (50 mg) and DMAP (1 mg) gave **10** as a solid (51mg, 65.3 % yield). ^{1}H NMR (700 MHz, $CDCl_3$) δ 8.52 (dd, *J* = 4.2, 1.7 Hz, 1H), 7.94 – 7.88 (m, 2H), 7.65 (s, 1H), 7.38 (d, *J* = 7.4 Hz, 1H), 7.31 – 7.27 (m, 1H), 7.19 (t, *J* = 5.6 Hz, 2H), 6.31 (dd, *J* = 22.9, 2.6 Hz, 2H), 6.15 (s, 1H), 3.86 (s, 3H), 3.63 (s, 1H), 1.82 – 1.63 (m, 4H), 1.27 (d, *J* = 2.5 Hz, 3H). ^{13}C NMR (176 MHz, $CDCl_3$) δ 165.8, 159.5, 145, 144.4, 136.5, 135.4, 135.0, 130, 128.1 124.8, 122.8, 122, 121.6, 120, 112.1, 97.0, 91.9, 55.3, 48, 39.6, 34.2, 26.8, 20.6. HRMS (EI) m/z Calcd. for $C_{24}H_{27}N_4O_2$ $[M+H]^+$: 403.2134, found 403.2171.

(S)-4-(1H-indol-3-yl)-N-(4-((6-methoxyquinolin-8-yl)amino)pentyl)butanamide (11): Following the general procedure 4-(1H-indol-3-yl) butanoic acid (32 mg), EDCI.HCl (32 mg.), dry CH_2Cl_2 (2 mL), S-Primaquine (50 mg) and DMAP (1 mg) gave **11** as a solid (59 mg, 68.6 % yield). ^{1}H NMR (700 MHz, $CDCl_3$) δ 8.52 (dd, *J* = 4.2, 1.6 Hz, 1H), 8.15 (s, 1H), 7.93 (dd, *J* = 8.2, 1.7 Hz, 1H), 7.57 (d, *J* = 8.9 Hz, 1H), 7.32 (d, *J* = 8.2 Hz, 1H), 7.30 (dd, *J* = 8.2, 4.2 Hz, 1H), 7.16 (t, *J* = 7.6 Hz, 1H), 7.08 (t, *J* = 7.4 Hz, 1H), 6.94 (d, *J* = 2.4 Hz, 1H), 6.34 (d, *J* = 2.5 Hz, 1H), 6.29 (d, *J* = 2.7 Hz, 1H), 5.98 (s, 1H), 5.39 (d, *J* = 5.8 Hz, 1H), 3.88 (s, 3H), 3.61 (s, 1H), 3.26 – 3.16 (m, 2H), 2.77 (t, *J* = 7.7 Hz, 2H), 2.16 (t, *J* = 7.5 Hz, 2H), 2.05 – 2.00 (m, 2H), 1.68 – 1.55

(m, 4H), 1.28 (d, *J* = 6.3 Hz, 3H). ^{13}C NMR (176 MHz, $CDCl_3$) δ 173.1, 159.5, 145, 144.4, 136.4, 135.4, 135, 130, 127.6, 122, 119.2, 118.9, 115.6, 111.2, 96.9, 91.8, 55.3, 47.96 ,39.5, 36.3, 34.1, 26.3, 26, 24.6, 20.7. HRMS (EI) m/z Calcd. for $C_{27}H_{33}N_4O_2$ $[M+H]^+$: 445.2604, found 445. 2622.

^{1}H (700 MHz, $CDCl_3$) and ^{13}C (175 MHz, $CDCl_3$) NMR spectrum of Compound 1

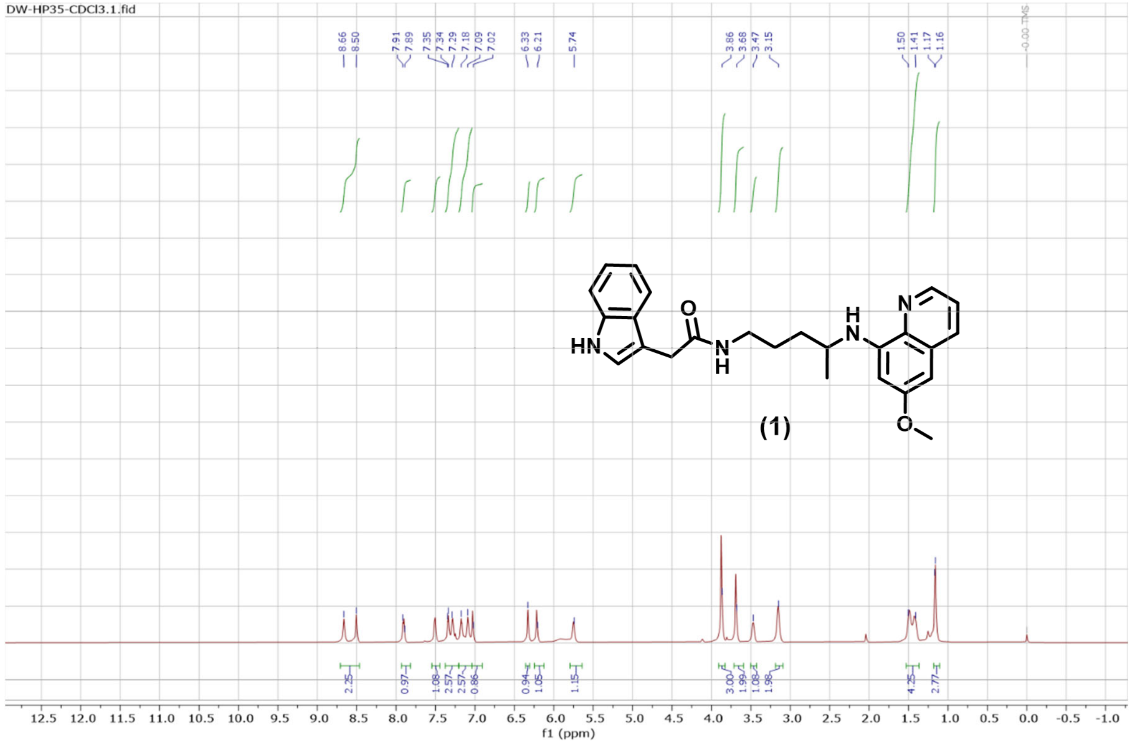

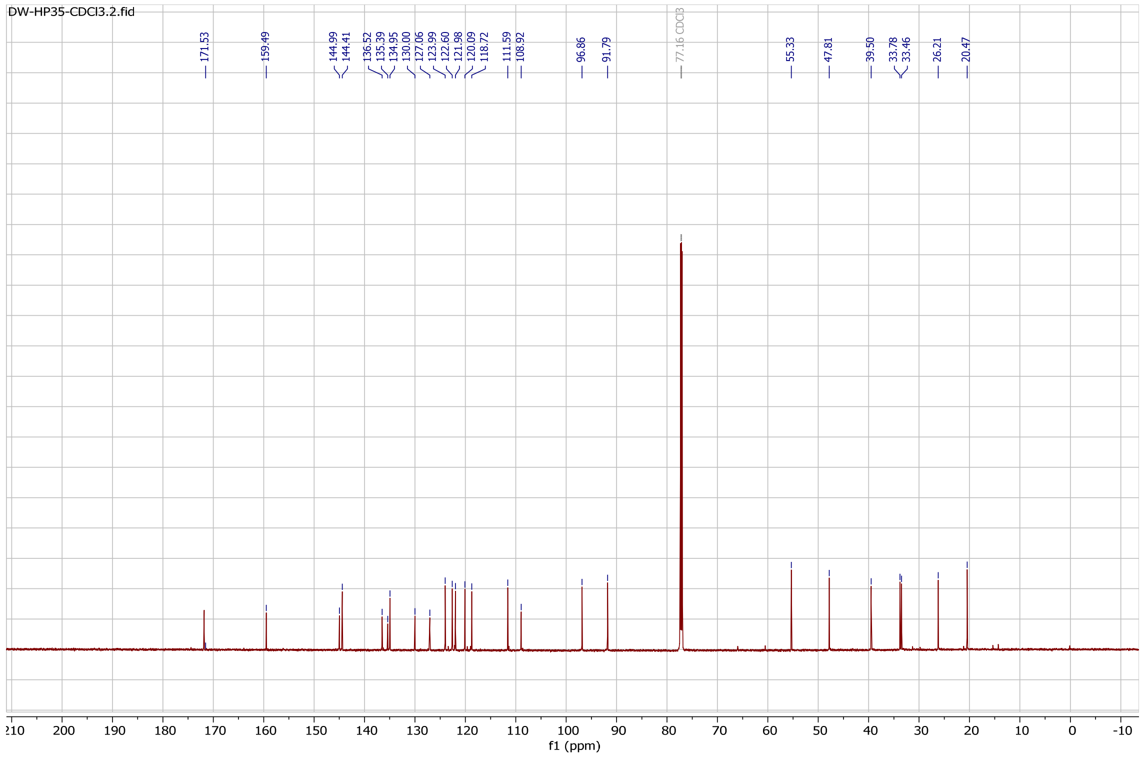

^{1}H (700 MHz, $CDCl_3$) and ^{13}C (175 MHz, $CDCl_3$) NMR spectrum of Compound 2

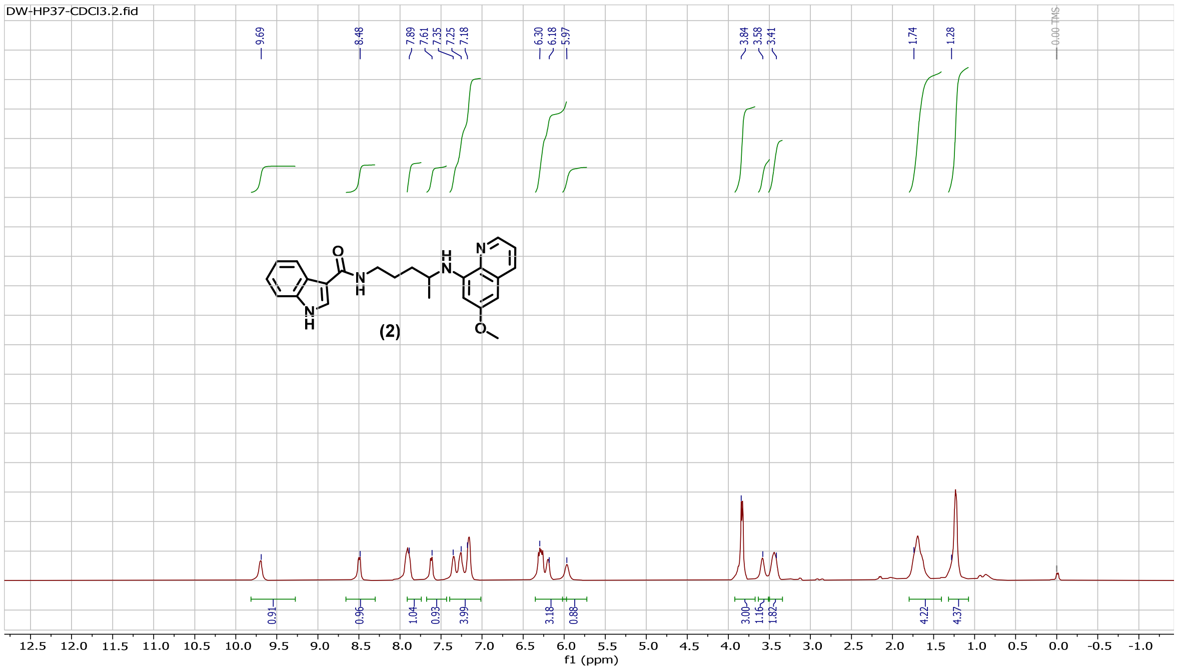

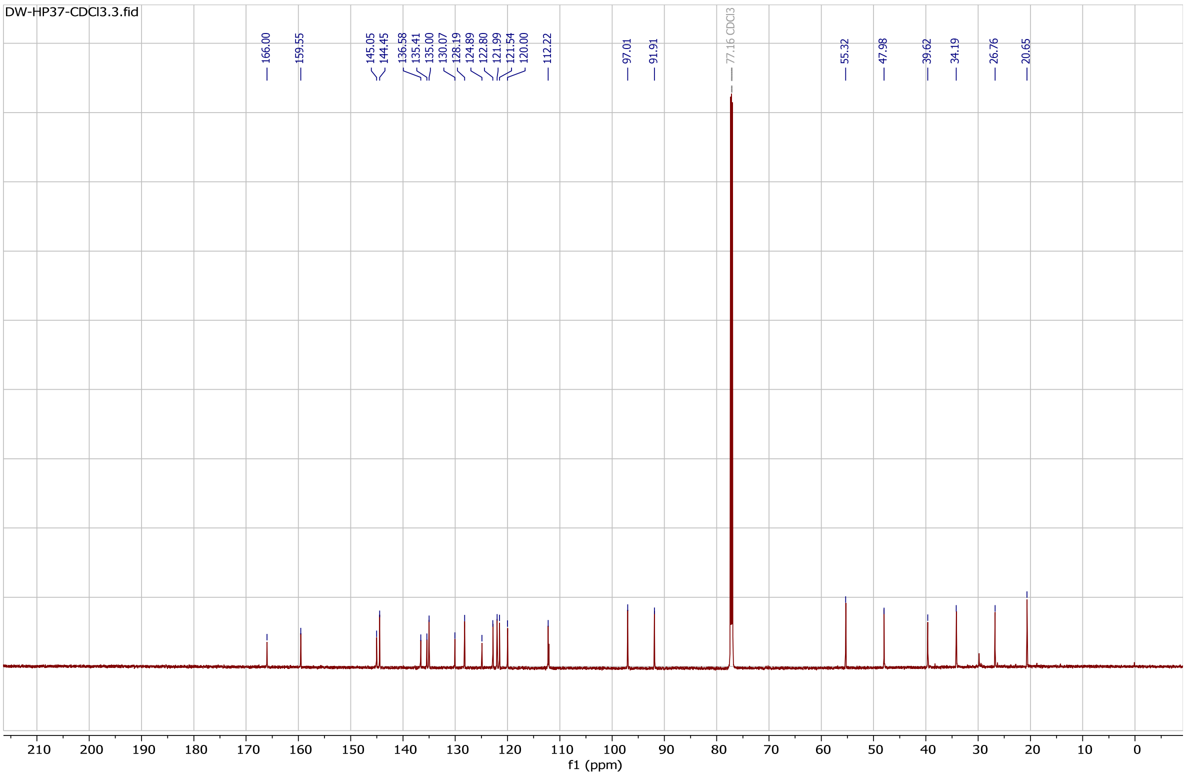

^{1}H (700 MHz, DMSO-*d6*) and ^{13}C (175 MHz, DMSO-*d6*) NMR spectrum of Compound 3

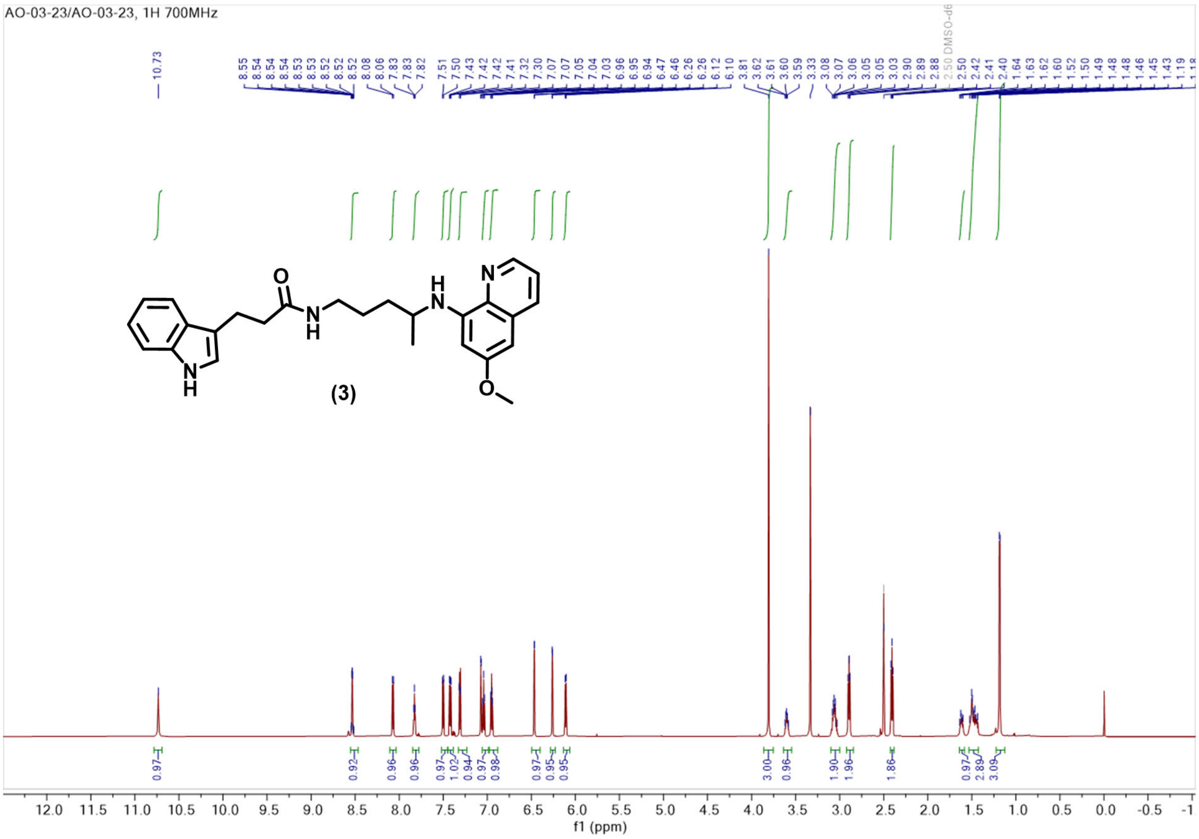

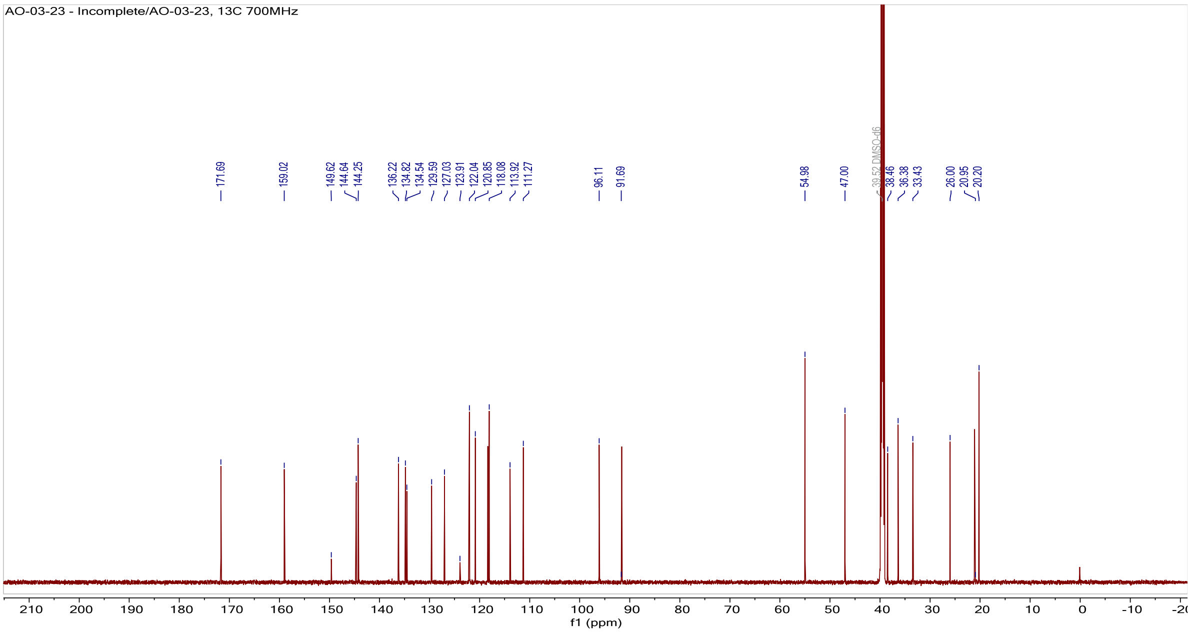

^{1}H (400MHz, $CDCl_3$) and ^{13}C (101 MHz, $CDCl_3$) NMR spectrum of Compound 4

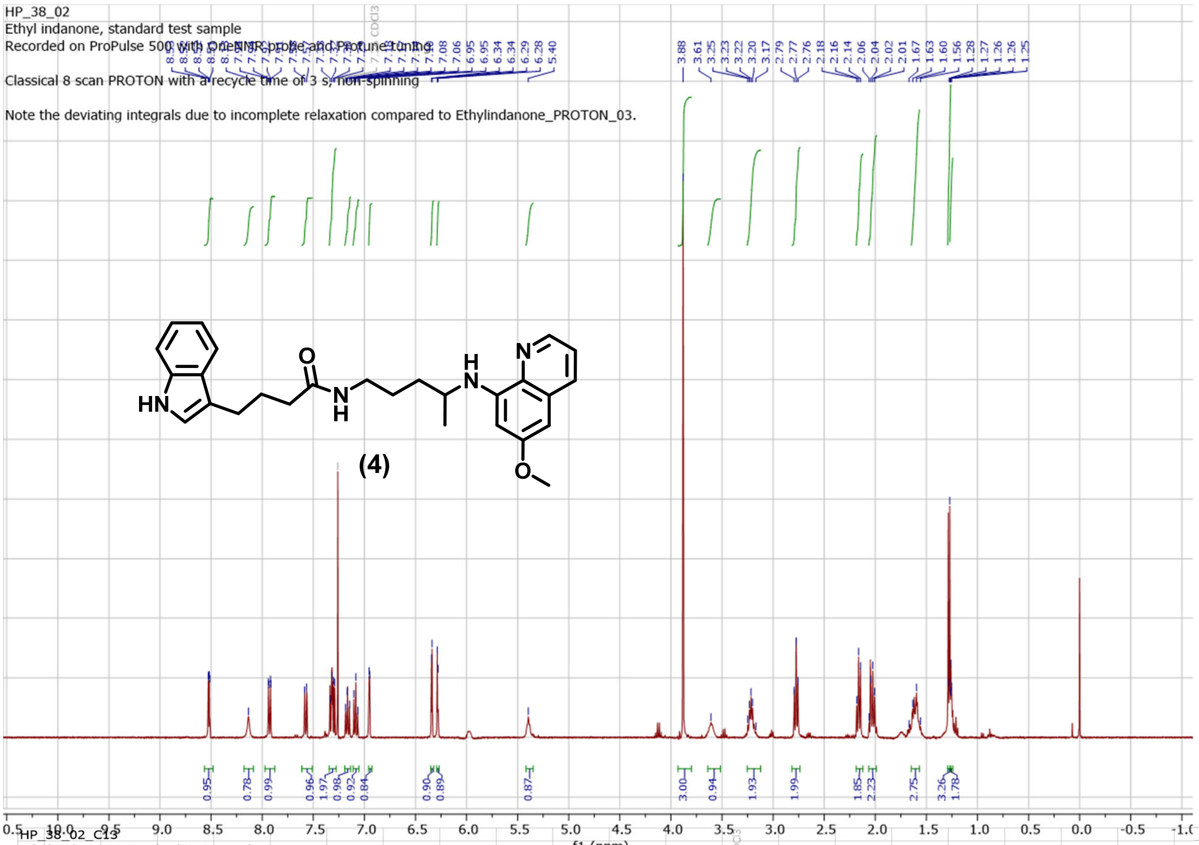

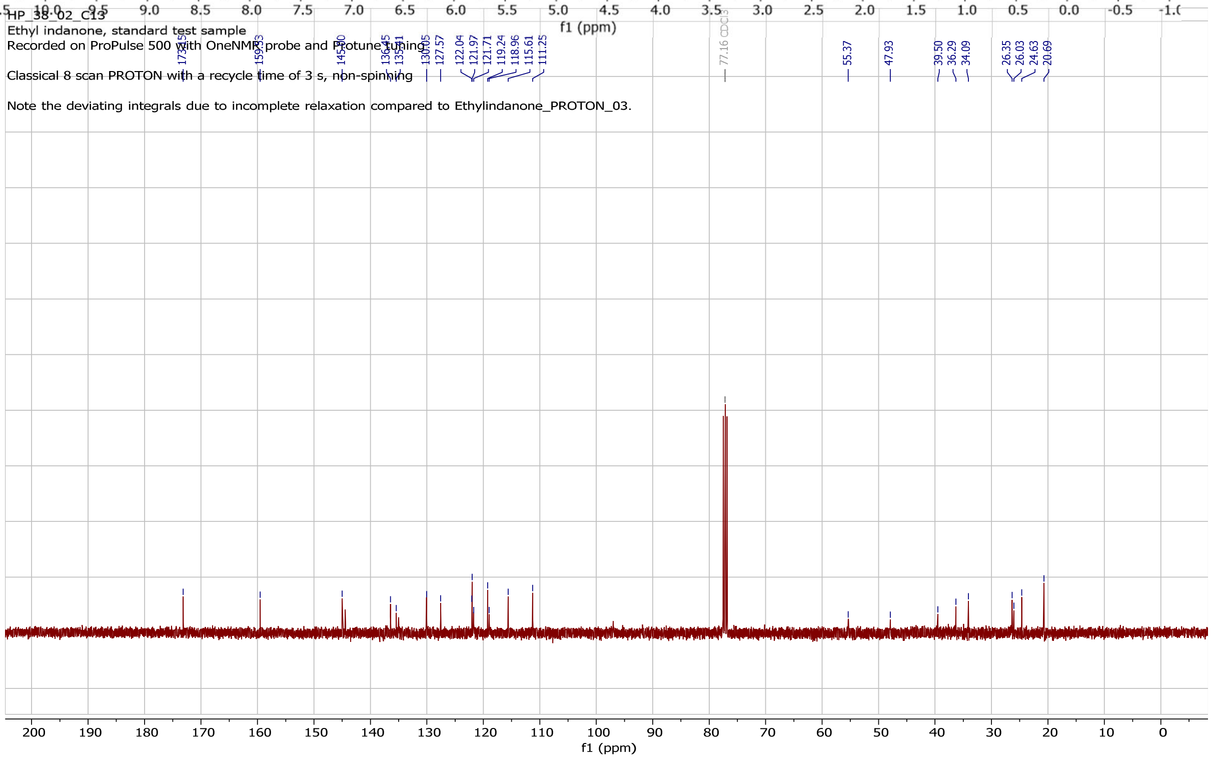

^{1}H (700 MHz, $CDCl_3$) and ^{13}C (175 MHz, $CDCl_3$) NMR spectrum of Compound 5

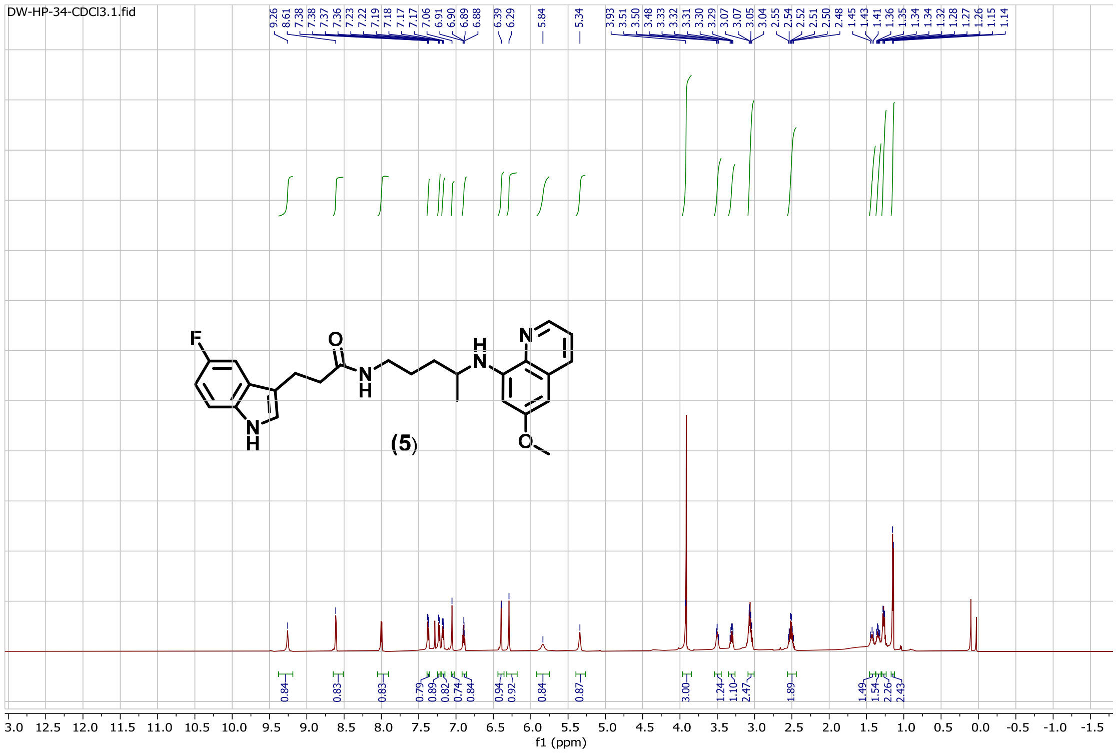

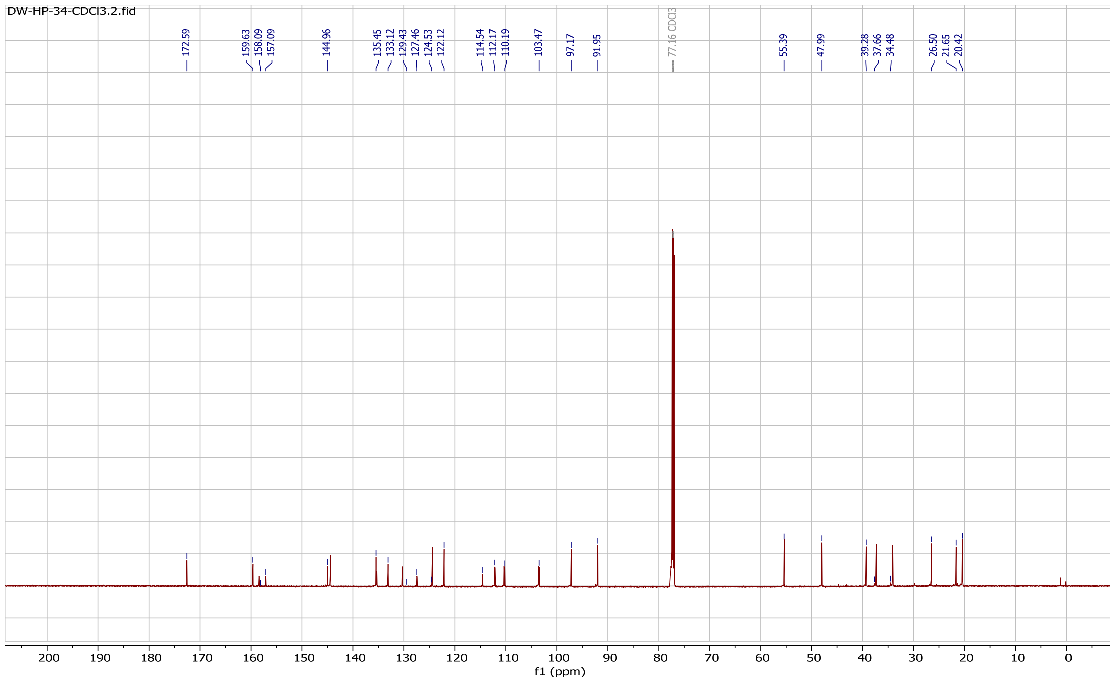

^{1}H (700 MHz, $CDCl_3$) and ^{13}C (175 MHz, $CDCl_3$) NMR spectrum of Compound 6

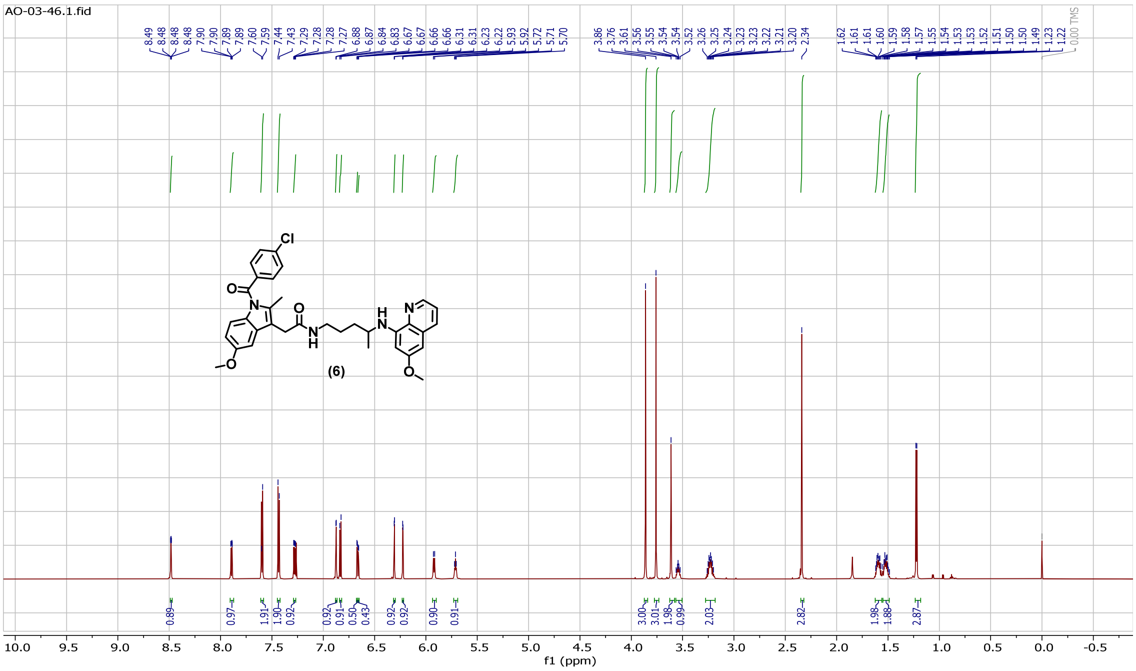

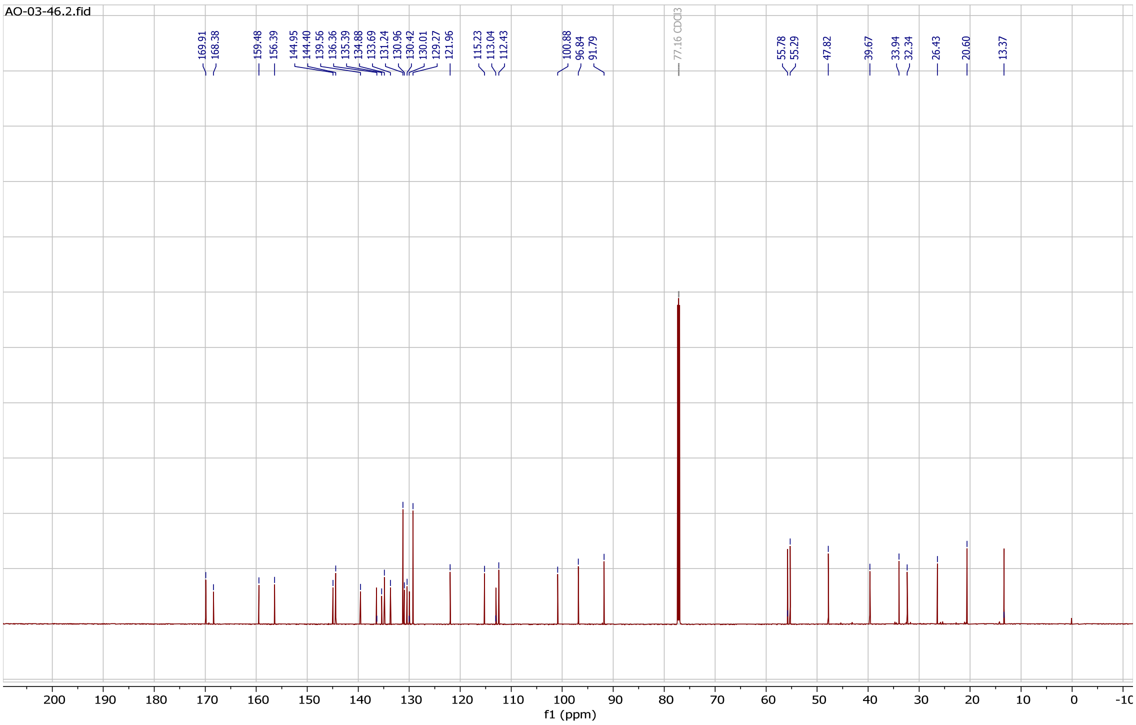

^{1}H (700 MHz, $CDCl_3$) and ^{13}C (175 MHz, $CDCl_3$) NMR spectrum of Compound 7

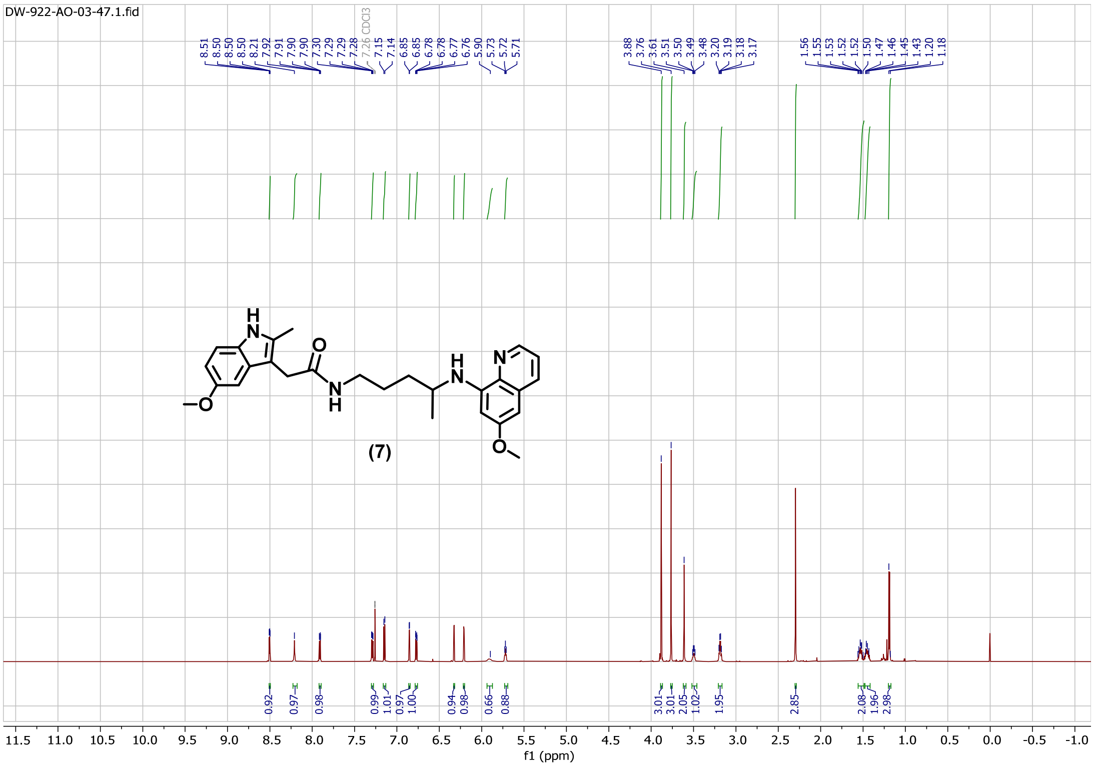

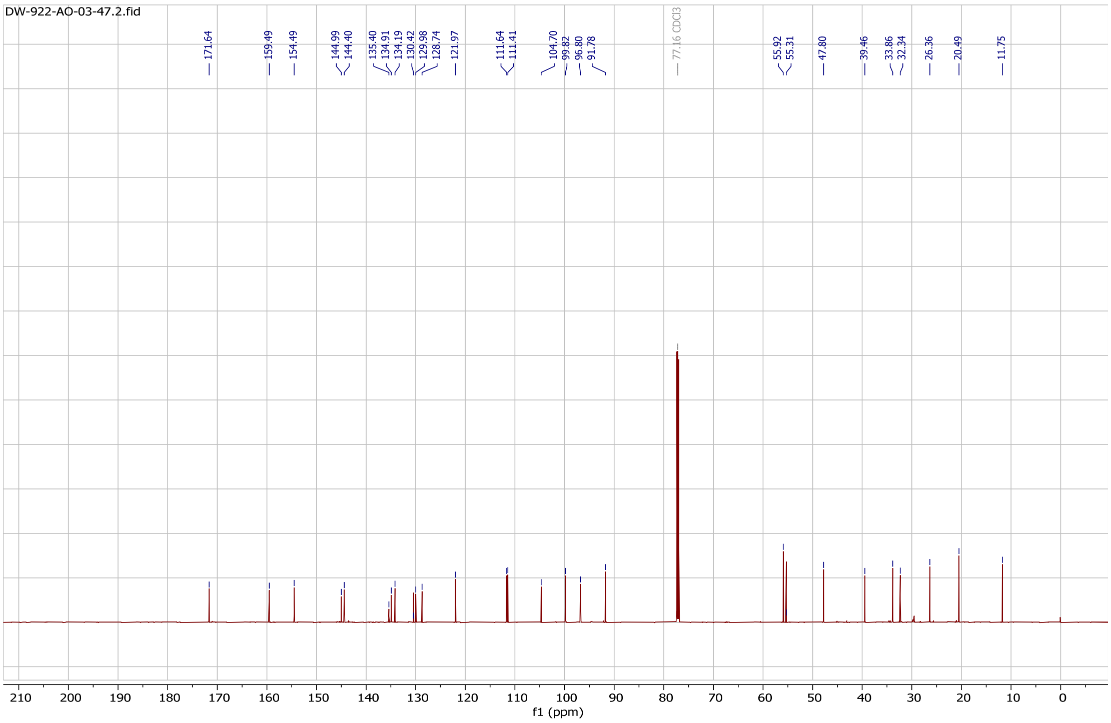

^{1}H (700 MHz, $CDCl_3$) and ^{13}C (176 MHz, $CDCl_3$) NMR spectrum of Compound 8

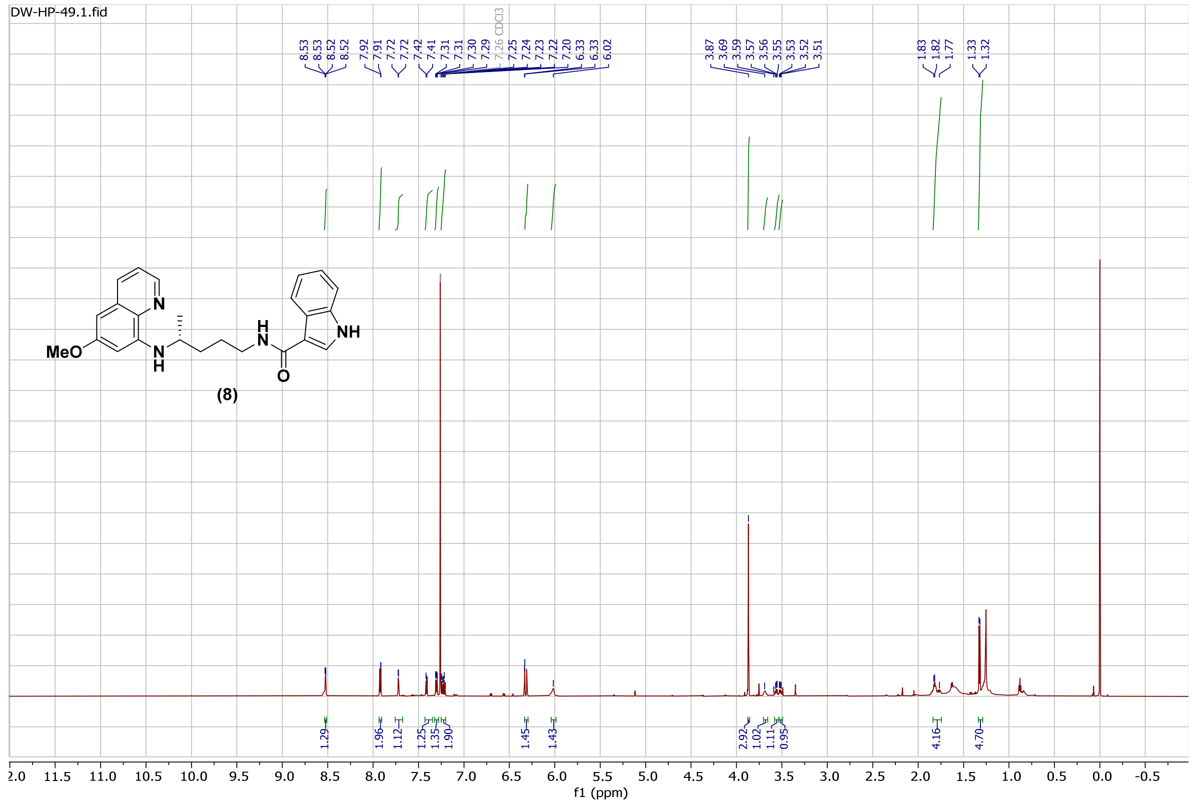

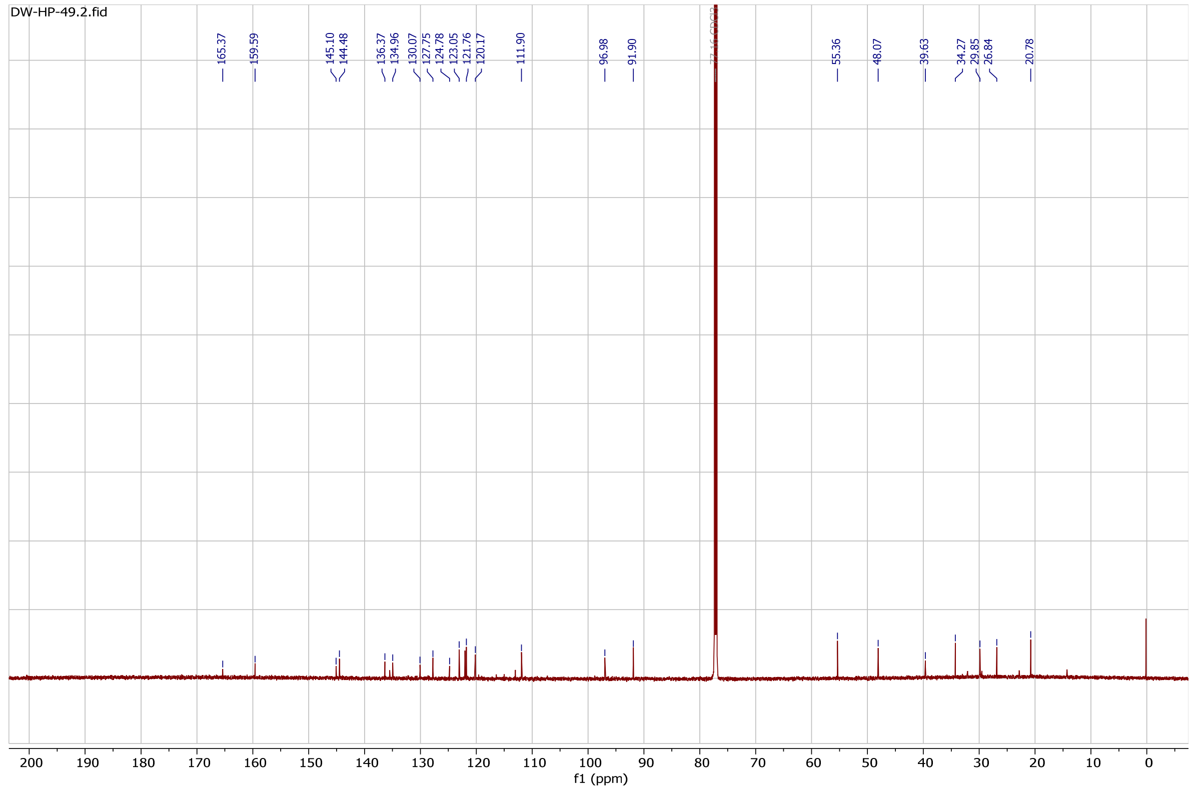

^{1}H (700 MHz, $CDCl_3$) and ^{13}C (176 MHz, $CDCl_3$) NMR spectrum of Compound 9

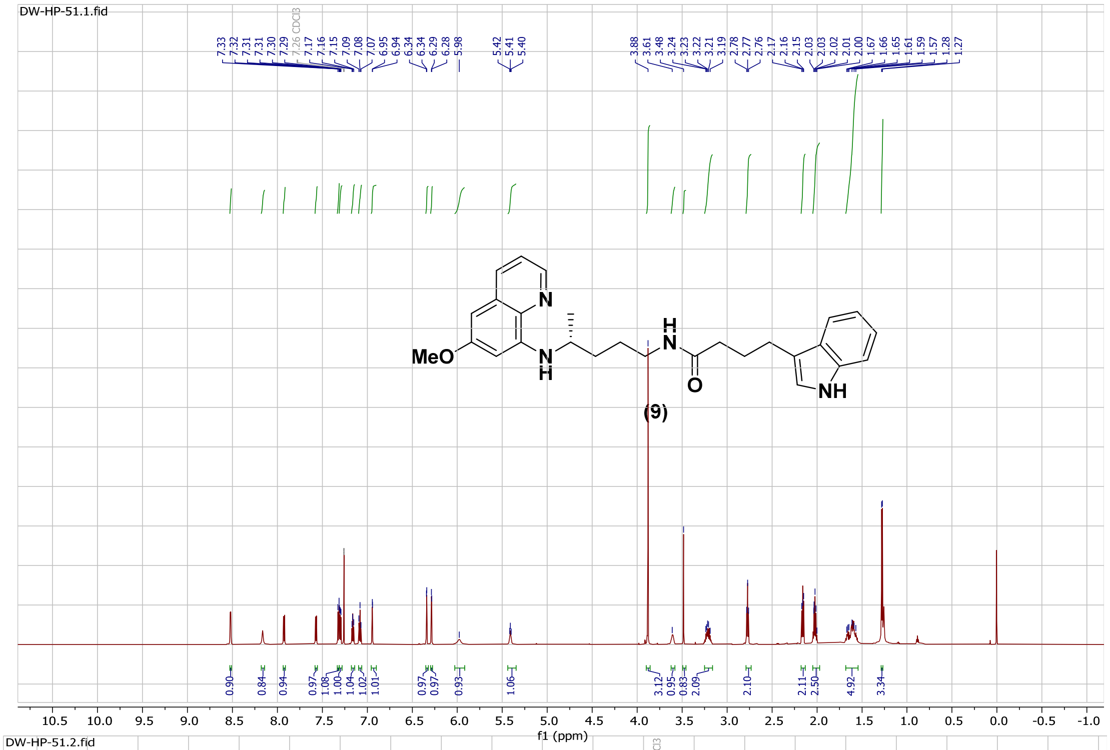

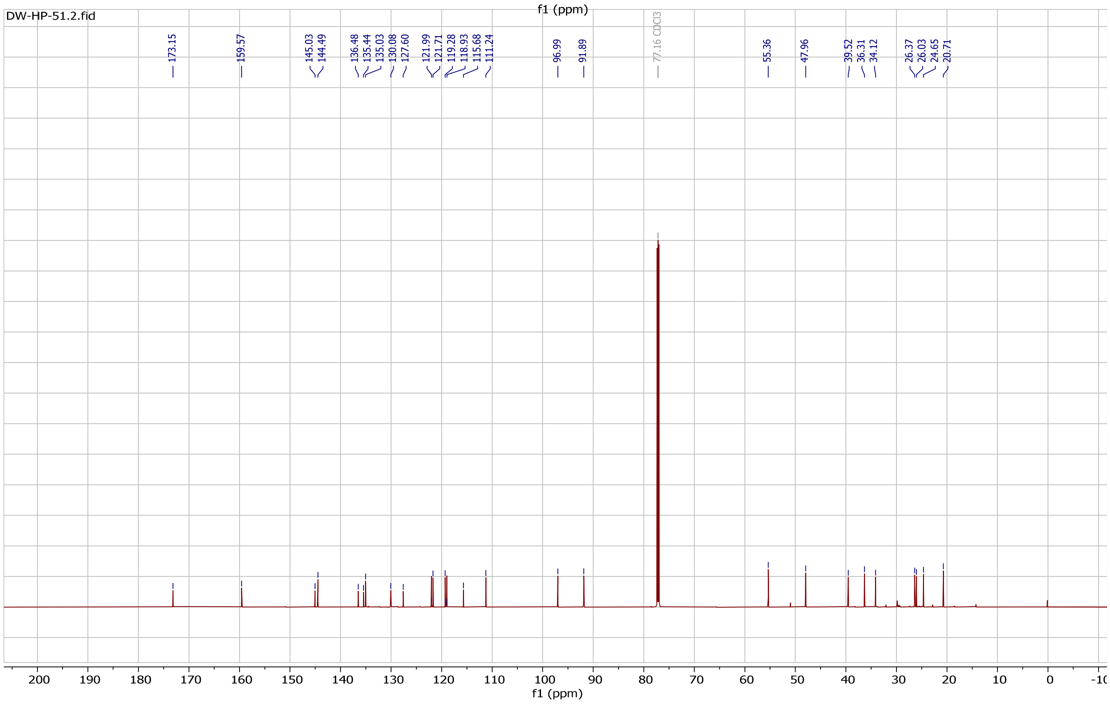

^{1}H (700 MHz, $CDCl_3$) and ^{13}C (176 MHz, $CDCl_3$) NMR spectrum of Compound 10

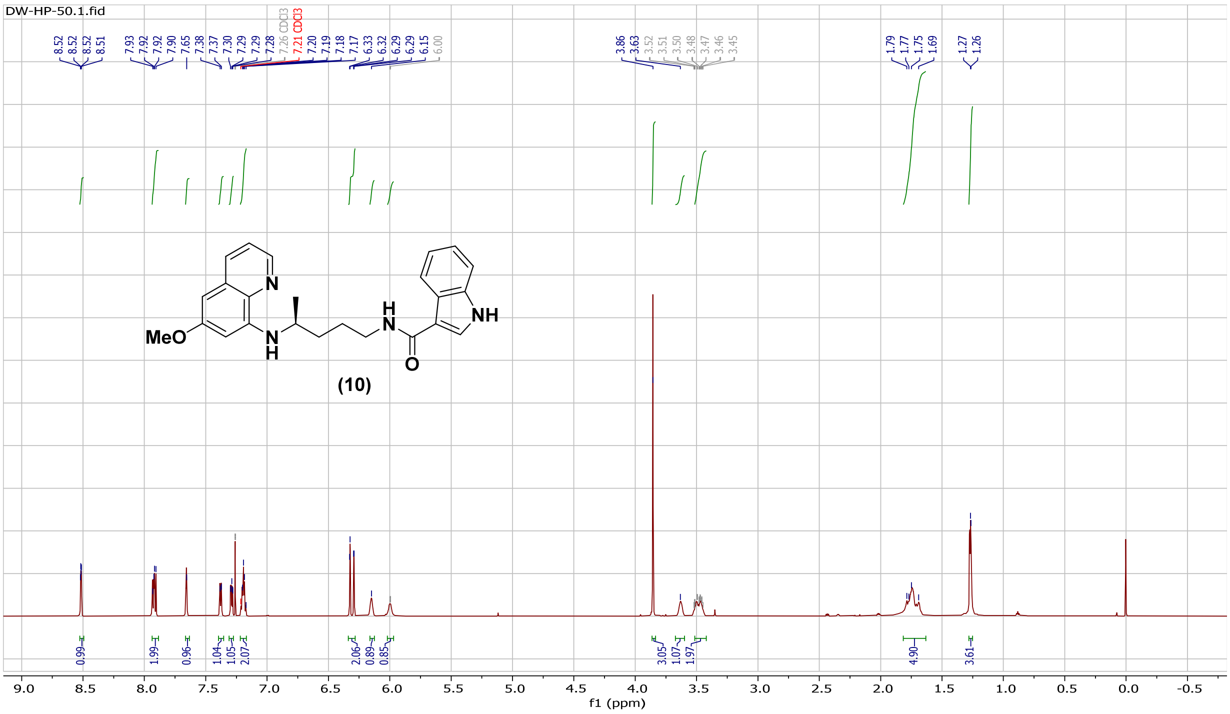

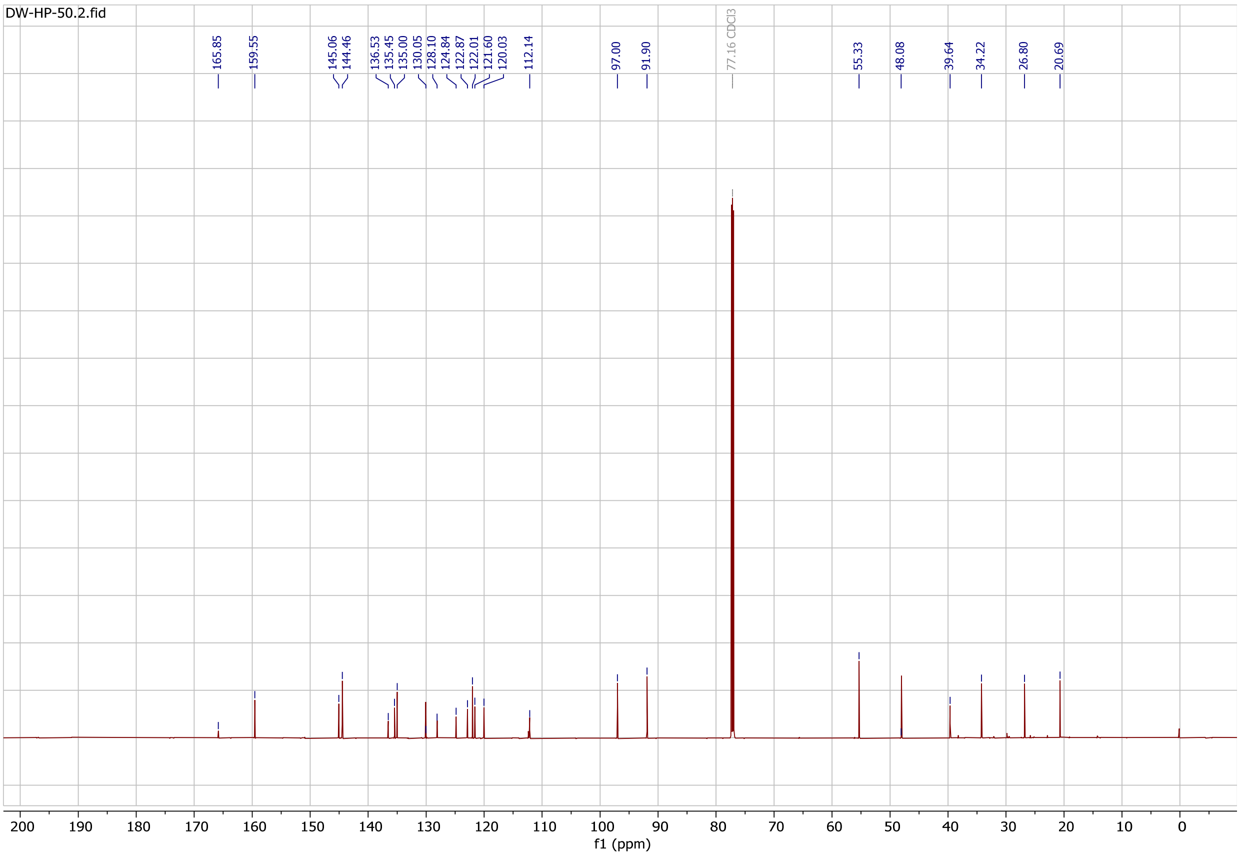

^{1}H (700 MHz, $CDCl_3$) and ^{13}C (176 MHz, $CDCl_3$) NMR spectrum of Compound 11

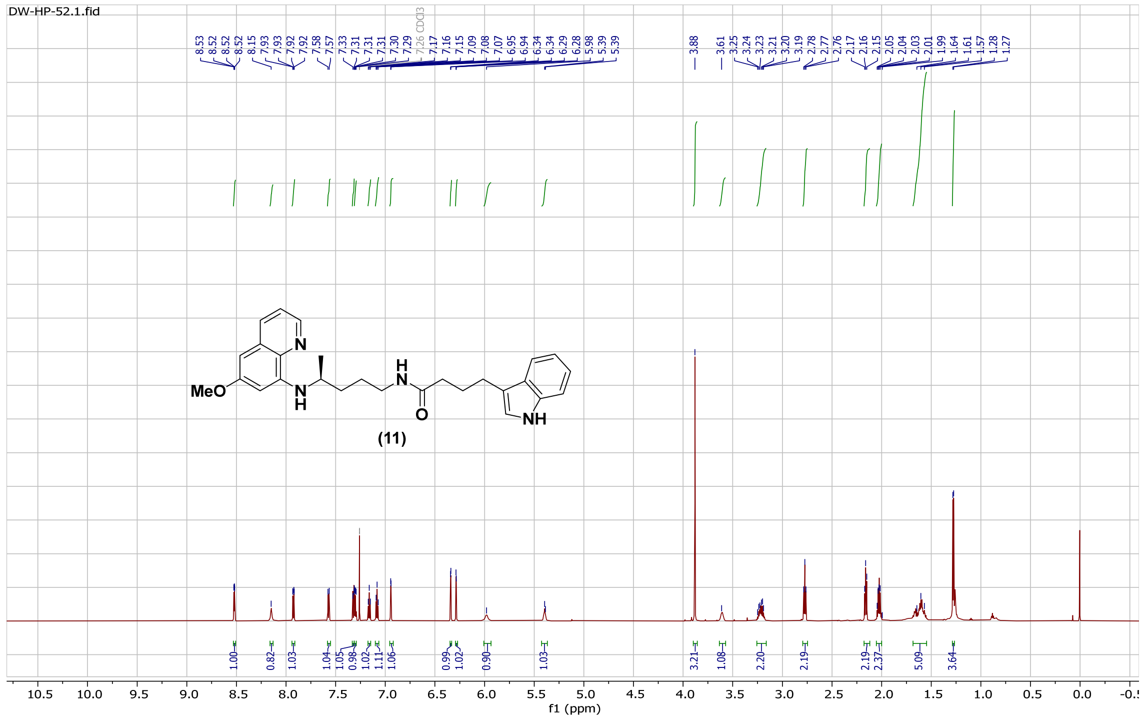

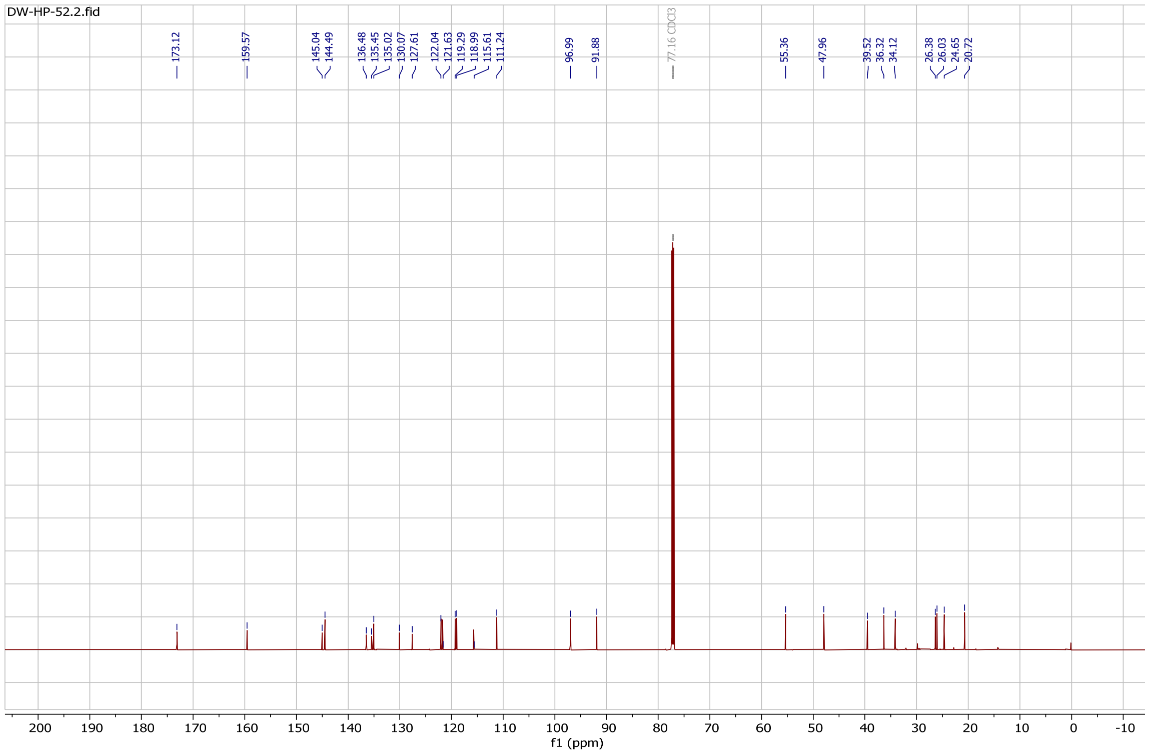

Supplement: Supplementary file 1 [file molecules-30-03988-s001.zip › molecules-3885527-supplementary.pdf]
